# The Combination of a BCL-xL PROTAC and an mTOR Inhibitor Sensitizes Pancreatic Ductal Adenocarcinoma to KRAS^G12D^ Inhibitor Treatment

**DOI:** 10.3390/cancers18060920

**Published:** 2026-03-12

**Authors:** Javed Miyan, Vignesh Vudatha, Lin Cao, Peiyi Zhang, Guangrong Zheng, Lei Zheng, Jose Trevino, Daohong Zhou, Sajid Khan

**Affiliations:** 1 Department of Biochemistry & Structural Biology, UT Health San Antonio, The University of Texas at San Antonio, 7703 Floyd Curl Drive, San Antonio, TX 78229, USA; 2 Department of Surgery, Virginia Commonwealth University School of Medicine, Richmond, VA 23298, USA; 3 Department of Medicinal Chemistry, College of Pharmacy, University of Florida, Gainesville, FL 32610, USA; 4 Mays Cancer Center, UT Health San Antonio, The University of Texas at San Antonio, 7703 Floyd Curl Drive, San Antonio, TX 78229, USA

**Keywords:** pancreatic ductal adenocarcinoma (PDAC), KRAS G12D, BCL-xL, PROTAC, mTOR, apoptosis, Drug resistance

## Abstract

Background/Objectives: Pancreatic ductal adenocarcinoma (PDAC) is a highly aggressive cancer with a five-year survival rate of approximately 13%, partly because of limited treatment options and resistance to therapies. Although the recently discovered KRAS G12D inhibitor MRTX1133 has shown promising efficacy in preclinical models, its clinical efficacy as a single agent is expected to be limited, as is the case with KRAS G12C inhibitors. Therefore, in this study, we evaluated potential combination strategies to enhance the therapeutic effect of MRTX1133. We combined MRTX1133 with the BCL-xL proteolysis-targeting chimera (PROTAC) DT2216 and the mTOR inhibitor everolimus. Methods: The sensitization of MRTX1133 by the combination of DT2216 + everolimus was tested in KRAS G12D-mutant PDAC cell lines using colony formation and apoptosis assays. The effects of MRTX1133 and/or DT2216 + everolimus on KRAS signaling and BCL-2 family proteins were assessed by immunoblotting and/or RT-PCR. The functional roles of BIM/NOXA were elucidated via immunoprecipitation (IP) and siRNA knockdown. Triple combination efficacy was evaluated in AsPC1 parental and MRTX1133-resistant xenografts, with pharmacodynamic effects confirmed by immunoblotting and immunohistochemistry. Results: The triple combination leads to significantly greater colony growth inhibition and apoptosis induction as compared with single agents or two-drug combinations in multiple KRAS G12D-mutant PDAC cell lines. Mechanistically, MRTX1133 treatment increased BIM and decreased NOXA levels, and the combination of DT2216/everolimus simultaneously enhanced BIM release and stabilized NOXA. In vivo, DT2216/everolimus combination significantly potentiated the anti-tumor activity of MRTX1133 in the AsPC1 PDAC xenograft model. Furthermore, the triple combination effectively overcame acquired MRTX1133 resistance in vitro and in the AsPC1 xenograft model. Conclusions: Collectively, our findings suggest that the combination of DT2216/everolimus potentiates the anti-tumor efficacy of MRTX1133 associated with enhanced apoptosis induction and inhibition of compensatory survival signaling.

## Simple Summary

Pancreatic ductal adenocarcinoma (PDAC) is an aggressive cancer with poor survival and lacks effective treatments. MRTX1133, a new investigational drug targeting the KRAS G12D mutation, which is common in PDAC, shows promise in preclinical studies but is unlikely to be effective as a single agent. In this study, we combined MRTX1133 with a BCL-xL-targeting degrader (DT2216) and the mTOR inhibitor everolimus for enhanced therapeutic efficacy. The triple combination more effectively killed KRAS G12D-mutant PDAC cells in culture and slowed tumor growth in mice than MRTX1133 alone, which was associated with simultaneously strengthening pro-death signals and blocking survival mechanisms in cancer cells. Notably, the triple combination also showed efficacy against mouse tumors that developed resistance to MRTX1133. These findings suggest that combining MRTX1133 with DT2216 and everolimus could be a more effective treatment strategy for patients with KRAS G12D-mutant PDAC.

## Significance

KRAS inhibitors, including KRAS G12D inhibitor MRTX1133, are promising therapeutics for treating KRAS-mutant pancreatic ductal adenocarcinoma (PDAC), but drug resistance limits their efficacy. Our study reveals that simultaneous induction of apoptosis and inhibition of compensatory survival signaling with a combination of a BCL-xL PROTAC degrader and an mTOR inhibitor is associated with significantly enhanced efficacy of MRTX1133 in KRAS G12D-mutant PDAC models, without increasing overt toxicity under the assessed parameters (mouse body weights, blood cell counts, AST/ALT activities) in immunodeficient mice.

## 1. Introduction

Pancreatic ductal adenocarcinoma (PDAC) comprises >90% of pancreatic cancer cases, is highly aggressive, and has one of the worst prognoses among human cancers [1]. The recent discoveries of KRAS inhibitors (KRASi), including pan-KRAS inhibitors and allele-specific KRAS inhibitors (including KRAS G12C inhibitors (G12Ci) and KRAS G12D inhibitors (G12Di)), have sparked renewed optimism for treating PDAC patients whose tumors harbor KRAS mutations. Among these inhibitors, G12Di MRTX1133 is a promising treatment for many PDAC patients [2,3,4,5,6]. This is because G12D mutations occur in ~45% of PDAC patients, and are associated with poorer prognosis compared to non-KRAS oncogenic drivers [7]. Though MRTX1133 is yet to be evaluated in clinical trials, its clinical efficacy as a monotherapy in PDAC is likely to be limited, as observed with G12Ci, due to a myriad of intrinsic, adaptive, and acquired resistance mechanisms [8,9,10,11]. Among these mechanisms, epithelial-to-mesenchymal transition (EMT) has been previously reported as a key contributor to KRASi resistance, particularly in treatment-refractory PDAC models [12,13,14]. In addition, the demonstrated mechanisms of resistance to G12Ci, such as feedback and compensatory activation of receptor tyrosine kinase (RTK)-mediated ERK/MAPK and the PI3K/AKT signaling pathway, have also been shown to be involved in resistance to MRTX1133 in early preclinical studies [4,15,16,17]. Among the major effector pathways downstream of KRAS, mTOR represents a particularly strategic therapeutic node because it integrates upstream signals from the PI3K/AKT and ERK/MAPK cascades via regulators such as TSC2, thereby coordinating proliferative and survival outputs that can persist or re-emerge through feedback and parallel inputs even when KRAS itself is inhibited [18,19,20]. However, targeting effectors of these pathways, including mTOR, individually is insufficient to overcome KRASi resistance because multiple RTKs are involved in the reactivation of various downstream effectors. In addition, targeting a single RTK or MAPK effector in combination with KRASi causes short-lived responses due to redundancy in KRAS downstream signaling effectors [15,16,17]. Although simultaneous inhibition of multiple signaling nodes could potentially improve efficacy, such approaches are likely to increase normal-tissue toxicities, limiting their translational feasibility. Taken together, these considerations highlight the need for more effective and better-tolerated combinatorial strategies to enhance the initial activity of MRTX1133 and overcome emerging resistance.

We and others have recently shown that suboptimal cell death induced by G12Ci monotherapy, in part due to overexpression of BCL-xL, is a major barrier to their reduced efficacy and development of resistance [21,22]. BCL-xL is an important anti-apoptotic protein in the BCL-2 family and is significantly upregulated in human PDACs compared to normal pancreatic tissue [23]. Its expression is negatively correlated with PDAC patient survival and response to therapy [23]. However, targeting BCL-xL with conventional inhibitors causes thrombocytopenia, an on-target and dose-limiting toxicity [24,25,26]. To overcome thrombocytopenia, we have developed DT2216, a platelet-sparing BCL-xL proteolysis-targeting chimera (PROTAC) degrader by linking navitoclax (a BCL-xL/BCL-2 dual inhibitor) with a ligand for Von Hippel-Lindau (VHL) E3 ligase. DT2216 targets BCL-xL for ubiquitination and proteasomal degradation [27]. Because VHL is minimally expressed in platelets but abundantly expressed in most tumor cells, DT2216 is highly potent against various tumor cell lines as a single agent or in combination with other antitumor agents, while having minimal antiplatelet activity compared to navitoclax [21,27,28,29,30,31]. Based on these preclinical findings, DT2216 has received orphan drug designation and fast-track designation from the FDA. DT2216 has completed a phase I clinical trial in patients with relapsed or refractory solid malignancies (NCT04886622), where it was generally well tolerated. Most patients experienced only transient thrombocytopenia with rapid recovery and demonstrated BCL-xL depletion in peripheral leukocytes [32]. Based on findings from this trial and promising preclinical results [30,33], DT2216 has advanced to a phase I/II clinical trial in combination with irinotecan for relapsed/refractory fibrolamellar carcinoma (NCT06620302) and a phase I trial in combination with paclitaxel for platinum-resistant ovarian cancer (NCT06964009).

In this study, we hypothesize that endogenous and MRTX1133-induced dysregulation of BCL-2 family proteins coupled with activation of compensatory survival signaling can contribute to intrinsic and acquired MRTX1133 resistance. Therefore, we aimed to develop a rational combination strategy to enhance the efficacy of MRTX1133 in PDAC. Specifically, we used DT2216 and everolimus (an FDA-approved mTOR inhibitor (mTOR-i)) to overcome intrinsic and acquired resistance to MRTX1133 by simultaneously augmenting tumor cell apoptosis and inhibiting compensatory survival signaling in G12D-mutated PDAC cell lines and xenograft models. Our findings suggest that MRTX1133 induces apoptotic reprogramming by upregulating pro-apoptotic BIM and downregulating pro-apoptotic NOXA. Furthermore, these reprogrammed cells are vulnerable to DT2216 and/or everolimus, inducing apoptosis via BCL-xL degradation-mediated BIM release, and NOXA upregulation, respectively. These agents work better in combination to improve the antitumor efficacy of MRTX1133, not only de novo but also in G12Di-resistant PDAC in vitro and in vivo.

## 2. Materials and Methods

### 2.1. Cell Lines and Cell Culture

Human PDAC cell lines (AsPC1 (RRID: CVCL_0152), HPAC (RRID: CVCL_3517), SW1990 (RRID: CVCL_1723), PANC1 (RRID: CVCL_0480), and BxPC3 (RRID: CVCL_0186)) were purchased from the American Type Culture Collection (ATCC, Manassas, VA, USA). AsPC1, SW1990, and BxPC3 cells were cultured in RPMI-1640 medium (Cat #22400–089, Thermo Fisher, Waltham, MA, USA). PANC1 cells were cultured in Dulbecco’s modified Eagle’s medium (DMEM) (Cat #12430-062, Thermo Fisher). All culture media were supplemented with 10% heat-inactivated fetal bovine serum (FBS) (Cat #S11150H, Atlanta Biologicals, Oakwood, GA, USA), 1% penicillin-streptomycin (Pen-Strep) solution (Cat #15140122, Thermo Fisher). HPAC cells were cultured in DMEM/F-12 medium (Cat #11330032, Thermo Fisher) supplemented with 5% FBS, 0.002 mg/mL insulin (Cat #12585-014, Thermo Fisher), 0.005 mg/mL human transferrin (Cat #T8158, MilliporeSigma, Burlington, MA, USA), 40 ng/mL hydrocortisone (Cat #H6909, MilliporeSigma), 10 ng/mL mouse epidermal growth factor (Cat #CB-40010, Fisher Scientific) and 1% pen-strep. Cell lines were used for experiments within 10 passages after thawing from liquid nitrogen. All cultures were confirmed Mycoplasma-free using the MycoAlert Mycoplasma Detection Kit (Cat #LT07–318, Lonza, Basel, Switzerland). All cell lines were maintained in a humidified incubator at 37 °C and 5% CO_2_.

### 2.2. Chemical Compounds

For in vitro experiments, DT2216 was provided by Dr. Guangrong Zheng’s laboratory (University of Florida, Gainesville, FL, USA), which they synthesized according to the previously described protocol [27]. For in vivo experiments, research-grade DT2216 was kindly provided by Dialectic Therapeutics (Dallas, TX, USA). MRTX1133 (Cat #HY-134813) and everolimus (Cat #HY-10218) were purchased from MedChemExpress (Monmouth Junction, NJ, USA). For in vitro experiments, the compounds were dissolved in DMSO at a 10 mM stock solution. In vivo formulations are described in the xenograft study section.

### 2.3. Cell Viability Assays

The cells were harvested by trypsinization, counted, and then seeded in CellStar µClear white 96-well plates (Cat #5665-5098, USA Scientific, Ocala, FL, USA) at a density of 5 × 10^3^ cells per well in 100 µL of medium. After overnight incubation, the test agents were added to the plates in 100 µL of medium at 2× titrated concentrations. The PrestoBlue™ HS Cell Viability Reagent (Cat #P50201, Thermo Fisher) was used to measure cell viability according to the manufacturer’s protocol. Briefly, 20 µL of PrestoBlue™ HS Cell Viability Reagent was added directly to the medium in each well, and the plates were incubated at 37 °C for 10 min. The fluorescence intensity was recorded at 560 nm (excitation)/590 nm (emission) using Synergy Neo2 multimode plate reader (Biotek, Winooski, VT, USA).

### 2.4. Colony Formation Assays

The cells were harvested by trypsinization, counted, and then seeded into 6-well plates at a density of 2 × 10^3^  cells per well. After overnight incubation, the cells were treated with test agents for 10–14 days. At the end of treatment, the cells were washed with PBS, fixed by adding 1 mL of absolute methanol per well for 15 min, and then stained with a 0.1% crystal violet solution for 15 min. The plates were thoroughly washed with distilled water and air-dried at room temperature. The images were captured using the ChemiDoc MP Imaging System (Bio-Rad, Hercules, CA, USA). Thereafter, 1 mL of 10% acetic acid was added to each well to dissolve the stained colonies, and the absorbance was recorded at 590 nm as a direct measure of colony growth.

### 2.5. Annexin V/PI Staining and Flow Cytometric Analysis

The cells were harvested by trypsinization, including dead cells from the culture medium, and centrifuged at 300× *g* for 5 min at 4 °C. The cell pellet was washed twice with cold PBS and resuspended in 1× Annexin V Binding Buffer (Cat #422201, BioLegend, San Diego, CA, USA) at a concentration of 2.5 × 10^6^ cells per mL. Approximately 2.5 × 10^5^ cells in 100 µL suspension were transferred into flow cytometry tubes and then stained with Alexa Fluor 647-conjugated Annexin V (1:50, Cat # 640911, BioLegend) and propidium iodide (PI, 1 μg/mL). Samples were incubated for 30 min at room temperature in the dark and then diluted with 400 µL Annexin V Binding Buffer. Data acquisition was performed on a BD FACSCelesta flow cytometer (BD Biosciences, San Jose, CA, USA). Apoptotic cell populations (live, early apoptotic, late apoptotic/necrotic) were analyzed based on Annexin V and PI staining and quantified using FlowJo v11 software (Tree Star Inc.).

### 2.6. Immunoblotting

Cells were lysed in 1× RIPA buffer (Cat #BP-115DG, Boston Bio Products, Ashland, MA, USA) supplemented with 1% protease + phosphatase inhibitor cocktail (Cat #PPC1010, MilliporeSigma), and the assay was performed as described previously [27]. Briefly, an equal amount of proteins (~40 µg/lane) was loaded onto a precast gel and transferred onto PVDF membranes. The membranes were blocked with 5% (*w*/*v*) non-fat dry milk in TBS-T buffer, and subsequently probed with primary antibodies overnight at 4 °C. After washing with TBST, the membranes were incubated with horseradish peroxidase (HRP)-linked secondary antibody for 1 h at room temperature. Finally, the membranes were incubated with chemiluminescent HRP substrate (Cat #WBKLS0500, MilliporeSigma) and imaged using the ChemiDoc MP Imaging System. The densitometric analysis of immunoblots was performed using Image J software v1.53k (RRID: SCR_003070). The details of antibodies are provided in the Supplementary Table S1.

### 2.7. RNA Extraction and Quantitative Real-Time PCR

RNA was isolated from cells using the RNeasy Mini Kit (Cat #74106, Qiagen, Hilden, Germany). A total of 1 µg of RNA was converted into cDNA using a high-capacity cDNA reverse transcription kit (Cat #4368813, Applied Biosystems, Foster City, CA, USA) as per the manufacturer’s instructions. The mRNA expressions were quantified using pre-designed Taqman probes of *BCL2L11* (Assay #Hs00708019_s1) and *PMAIP1* (Assay #Hs00560402_m1) from Thermo Fisher. The expression of *GAPDH* (Assay #Hs02786624_g1) was used for normalization, and the level in untreated cells was used as the baseline. Fold change in gene expression was calculated using the ΔΔCT method.

### 2.8. Immunoprecipitation (IP)

Cells were lysed in the 1x Pierce IP lysis buffer (Cat #87787; Thermo Fisher) supplemented with 1% protease + phosphatase inhibitor cocktail, and the assay was performed as described previously [27]. The supernatants were collected and precleared by incubating with 1 µg of mouse anti-IgG (Cat #sc-2025; Santa Cruz Biotechnology, Dallas, TX, USA) and 20 µL of protein A/G-PLUS agarose beads (Cat #sc-2003; Santa Cruz Biotechnology) for 30 min at 4 °C. The supernatants containing 1 mg of protein were incubated with 2 µg of anti-BIM (Cat #sc-374358, Santa Cruz Biotechnology, RRID: AB_10987853), or 2 µg of anti-MCL-1 (Cat #sc-12756, Santa Cruz Biotechnology, RRID: AB_627915) or 2 µg of anti-IgG antibody (Cat # sc-2025, Santa Cruz Biotechnology, RRID: AB_737182) overnight followed by incubation with 25 µL protein A/G-PLUS agarose beads for 1 h at 4 °C. Thereafter, the protein A/G-PLUS agarose beads were collected by centrifugation, followed by 3x washing with IP lysis buffer, and then mixed with 50 µL of Laemmli’s SDS-buffer and boiled to release protein into the supernatant. The supernatant was then subjected to immunoblot analysis for BIM, BCL-xL, MCL-1, BAX, BAK, and NOXA. Total cell lysate (input) samples were run alongside. Anti-rabbit HRP-conjugated Fc fragment specific secondary antibody (Cat #111-035-046, RRID: AB_2337939, dilution 1:10,000, Jackson ImmunoResearch, West Grove, PA, USA) was used to detect immune complexes.

### 2.9. Small Interference (Si) RNA-Mediated Knockdown of PMAIP1 and BCL2L11

The siRNAs for human *PMAIP1* (Cat. No. L-005275-00-0005, Horizon Discovery, Cambridge, UK) and *BCL2L11* (Cat. No. L-004383-00-0005, Horizon Discovery) were reconstituted in RNase-free water at a stock concentration of 25 μM, and 25 nM of siRNA was transfected to the cells using DharmaFECT1 Transfection Reagent (Cat. No. T-2001-03, Horizon Discovery) as per the manufacturer’s instructions. The siGENOME non-targeting control siRNA (siCtrl) (Cat. No. D-001210-01-05; Horizon Discovery) was used as a negative control. After 48 h of transfection, cells were plated and treated for apoptosis analysis. Gene-knockdown efficiency was confirmed using Western blot analysis.

### 2.10. Generation of MRTX1133-Resistant Cells

AsPC1 cells were plated in a T-25 flask and initially exposed to 100 nM of MRTX1133. After the cells became resistant, the MRTX1133 concentration increased to 200 nM, then doubled, until the cells became resistant to 3.2 µM MRTX1133. Totally, it took two months for the cells to become resistant to 3.2 µM of MRTX1133. At this time, the cells were considered MRTX1133-resistant (referred to as AsPC1-MR) for our experiments and maintained at 3.2 µM MRTX1133.

### 2.11. Xenograft Study

SCID-Beige female mice aged 5–6 weeks were purchased from the Charles River Laboratories (Stock No. 250, Wilmington, MA, USA). AsPC1 cells mixed with 50% Matrigel (Cat #356237, Corning, Corning, NY, USA) and 50% plain RPMI-1640 medium (5 × 10^6^ cells/100 µL/mouse) were injected subcutaneously (s.c.) into the right flank region of mice as described previously [27]. Tumor growth was monitored daily, tumor size was measured twice a week with digital calipers, and tumor volume was calculated using the formula (Length × Width^2^ × 0.5). The mice were randomized into different treatment groups when their tumors reached 150 mm^3^ in volume. For the efficacy study, mice were treated with vehicle, MRTX1133 (3 mg/kg, twice a day (b.i.d.), 5 days ON/2 days OFF, i.p.), DT2216 (15 mg/kg, twice a week, i.p.), everolimus (2.5 mg/kg twice a week, p.o.) MRTX1133 + DT2216, MRTX1133 + Everolimus and MRTX1133 + DT2216 + Everolimus. MRTX1133 and Everolimus were dissolved individually in 10% DMSO (*v*/*v*) and 90% (*v*/*v*) of 20% (*w*/*v*) Captisol (Cat. No. NC0604701, Cydex Pharmaceuticals, a Ligand Company, San Diego, CA, USA). A CRO formulated DT2216. All the animal procedures were performed in accordance with the IACUC guidelines at the University of Texas Health Science Center at San Antonio.

MRTX1133-resistant xenograft model: Tumors were considered resistant when they exhibited significant regrowth following maximal regression after initial MRTX1133 treatment at 10 mg/kg, b.i.d., i.p., 7 days a week. Consistent tumor growth despite continued MRTX1133 exposure confirmed the establishment of acquired resistance in this model. At this point, mice were randomized into four different treatment groups, i.e., MRTX1133 (10 mg/kg, b.i.d., 7 days a week, i.p.), MRTX1133 (10 mg/kg, b.i.d., 7 days a week, i.p.) plus DT2216 (15 mg/kg, twice a week, i.p.), MRTX1133 (10 mg/kg, b.i.d., 7 days a week, i.p.) plus everolimus (2.5 mg/kg twice a week, p.o.), and MRTX1133 (10 mg/kg, b.i.d., 7 days a week, i.p.) plus DT2216 (15 mg/kg, twice a week, i.p.) plus everolimus (2.5 mg/kg twice a week, p.o.).

### 2.12. Pharmacodynamic (PD) Study

Tumor-bearing mice were treated with MRTX1133 b.i.d. for 5 days, two doses of DT2216, and everolimus at a 3-day interval, and the same dosage as used in the xenograft study. For the PD study, the treatment was started when the average tumor size reached ~400 mm^3^. The tumors were subsequently harvested 24 h after the last dose of DT2216. A portion of the tumors was flash-frozen for immunoblotting analysis, and another portion was fixed in 4% paraformaldehyde for histological analysis.

### 2.13. Hematoxylin & Eosin (H&E) Staining

H&E staining was performed using the Leica ST5010 Autostainer XL (Leica Biosystems, Deer Park, IL, USA). Tissue sections were deparaffinized by incubating at 60 °C for 30 min, followed by four washes in xylene (5 min each). Sections were then rehydrated through a graded ethanol series (100%, 95%, 80%, and 70%, 5 min each) and rinsed in distilled water (dH_2_O). For nuclear staining, sections were incubated in hematoxylin, rinsed in dH_2_O, differentiated in an acidic alcohol solution, blued in an alkaline solution, and rinsed again. Eosin was then applied for cytoplasmic staining. After staining, sections were dehydrated through graded ethanol (70% to 100%) and cleared in xylene (4×, 5 min each). Finally, slides were mounted using synthetic permount and a glass coverslip. Images were acquired using a Zeiss Axio Vert.A1 (Carl Zeiss Microscopy GmbH, Jena, Germany) inverted microscope under 10× magnification.

### 2.14. Immunohistochemistry (IHC)

IHC staining was performed using the Leica ST5010 Autostainer XL and the Dako EnVision FLEX system (Agilent, Santa Clara, CA, USA). Tissue sections were deparaffinized by heating at 60 °C for 30 min, followed by xylene washes (4×, 5 min each) and rehydration through a graded ethanol series (100% ethanol twice for 5 min each, 95% ethanol for 5 min, and 70% ethanol for 5 min). Heat-induced epitope retrieval was performed using Dako PT Link (Agilent Technologies, Glostrup, Denmark) with EnVision FLEX Target Retrieval Solution (low or high pH, Agilent Technologies) at 97 °C for 20 min. After retrieval, sections were washed in EnVision FLEX Wash Buffer (Agilent Technologies) and blocked with EnVision FLEX Peroxidase Block for 5 min. Primary antibody (Ki67 (Cat. No. M7240, RRID: AB_2142367, dilution 1:170, Agilent, Santa Clara, CA, USA) or cleaved caspase-3 (Cat. No. 9664, RRID: AB_2070042, dilution 1:600, Cell Signaling Technology, Danvers, MA, USA)) incubation was performed at an optimized concentration and duration, followed by extensive washing. Sections were then incubated with FLEX/HRP-labeled polymer for 30 min, washed, and developed using DAB+ Substrate-Chromogen for 5 min. Counterstaining was performed with Biocare CAT Hematoxylin for 5 s, followed by bluing for 30 s. Finally, sections were washed, dehydrated, cleared, and cover-slipped for microscopic analysis. Images were acquired using a Zeiss Axio Vert.A1 inverted microscope under 10× magnification.

### 2.15. Statistical Analysis

For the analysis of means of three or more groups, analysis of variance (ANOVA) tests were performed. If ANOVA justified post hoc comparisons between group means, the comparisons were conducted using Dunnett’s multiple-comparison test for comparing each group with the control group or Tukey’s multiple-comparison test for comparing each group with every other group. A two-sided unpaired Student’s *t*-test was used to compare the means of two groups. *p* < 0.05 was considered to be statistically significant.

## 3. Results

### 3.1. The Combination of DT2216 and Everolimus Potentiates the Anti-Clonogenic Activity of MRTX1133 and Promotes Apoptosis Induction In Vitro

**Figure 1:**
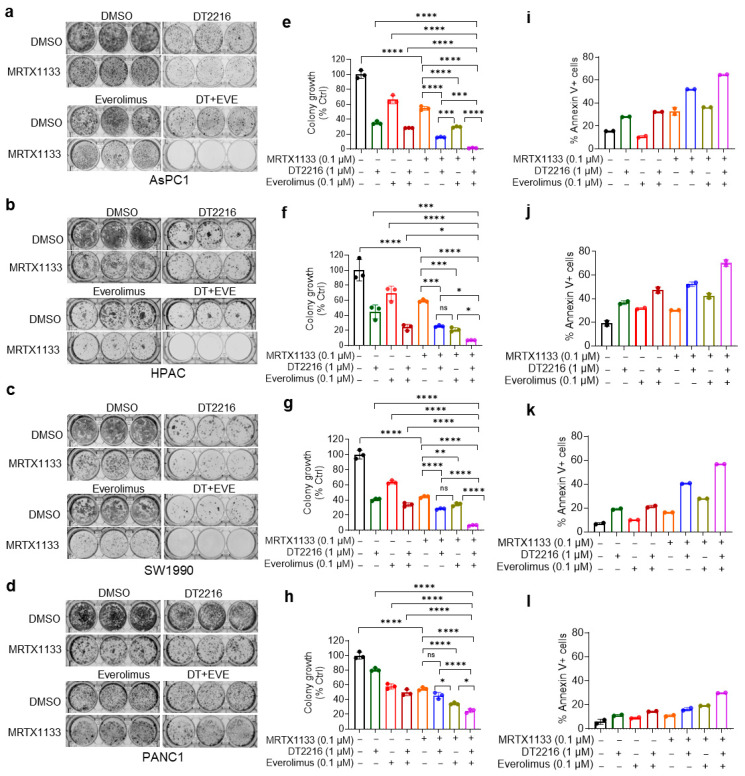
The combination of DT2216 and everolimus enhances the anti-clonogenic effect of MRTX1133 and promotes apoptosis. (**a**–**d**) Cell colony images of AsPC1 (**a**), HPAC (**b**), SW1990 (**c**), and PANC1 (**d**) after treatment with MRTX1133, DT2216, everolimus, and their combinations as indicated for 10–14 days, followed by crystal violet staining. (**e**–**h**). Colorimetric measurement of colony growth in AsPC1 (**e**), HPAC (**f**), SW1990 (**g**), and PANC1 cells (**h**). Data are presented as mean ± SD (n = 3 cell culture replicates). * *p* < 0.05, ** *p* < 0.01, *** *p* < 0.001, **** *p* < 0.0001, ^ns^ *p* > 0.05, i.e., not significant as determined by one-way ANOVA and Tukey’s multiple comparisons test. (**i**–**l**). Percentage annexin V^+^ apoptotic cell population in AsPC1 (**i**), HPAC (**j**), SW1990 (**k**), and PANC1 (**l**) after treatment with MRTX1133, DT2216, everolimus, and their combinations at indicated concentrations for 48 h. Data are presented as mean ± SD (n = 2 independent experiments).

We performed long-term colony formation assays to test the combinations of DT2216/everolimus with MRTX1133 in AsPC1, HPAC, SW1990, and PANC1 cells. All three compounds, as single agents, had significant but partial inhibition of colony growth. MRTX1133 alone caused 40% to 60% reduction in colony growth compared to the DMSO control. The addition of DT2216 or everolimus to MRTX1133 significantly reduced colony formation compared to MRTX1133 alone. More importantly, the triple combination of MRTX1133+DT2216+everolimus was significantly more potent than two-drug combinations and led to nearly complete inhibition of colony growth in AsPC1, HPAC, and SW1990 cell lines at the tested concentrations (Figure 1a–c,e–g). Compared with the effects in the other three cell lines, the triple combination had a less pronounced effect in PANC1 cells, yet it was significantly greater than that of the individual agents or two-drug combinations (Figure 1d,h).

Next, we performed flow cytometric analysis to quantify apoptosis after labelling cells with annexin V and PI. The results indicate modest apoptosis induction by MRTX1133 alone, which was enhanced when it was combined with DT2216 or everolimus, and the highest apoptosis induction was obtained with the triple combination (Figure 1i–l). The results from apoptosis analysis were also correlated with those obtained with colony formation assays. In addition, we measured cellular apoptosis by immunoblotting analysis of cleaved caspase-3 (CC3) and cleaved PARP. MRTX1133 alone induced minimal apoptosis as indicated by barely detectable CC3 and cleaved PARP levels. However, the addition of DT2216 leads to a substantial increase in both CC3 and cleaved PARP, which was further increased after the triple combination treatment (Supplementary Figure S1a–c). Pretreatment with the pan-caspase inhibitor Q-VD-OPh (QVD) substantially reduced apoptosis induced by the triple combination (Supplementary Figure S1d), indicating that the apoptosis induction is caspase-dependent. Overall, MRTX1133, when combined with DT2216 and everolimus, reduces clonogenic growth and is associated with increased caspase-dependent apoptosis in short-term assays.

### 3.2. MRTX1133 Increases Pro-Apoptotic BH3-Only Protein BIM and Decreases NOXA in G12D-Mutated PDAC Cells

**Figure 2:**
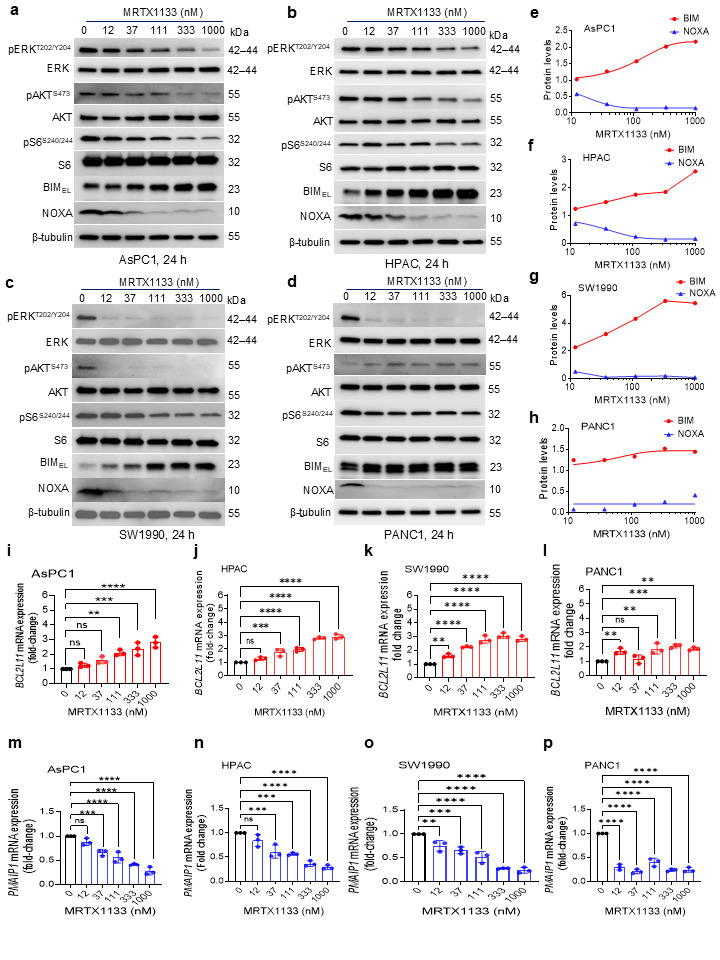
MRTX1133 increases BIM and decreases NOXA expression in G12D-mutated PDAC cells. (**a**–**d**). Immunoblot analyses of phosphorylated (p)- and total- ERK, AKT, and S6, BIM extra-long isoform (BIM_EL_), and NOXA in AsPC1 (**a**), HPAC (**b**), SW1990 (**c**), and PANC1 (**d**) cell lines after treatment with indicated concentrations of MRTX1133 for 24 h. The β-tubulin was used as an equal loading control. (**e**–**h**). Densitometric analysis of BIM_EL_ and NOXA immunoblots in AsPC1 (**e**), HPAC (**f**), SW1990 (**g**), and PANC1 (**h**). Immunoblots detect three isoforms of BIM, i.e., short isoform (BIM_S_), long isoform (BIM_L_), and extra-long isoform (BIM_EL_). Among them, BIM_EL_ is the major isoform and is shown here. (**i**–**l**). mRNA expression levels of BIM-coding gene *BCL2L11* in AsPC1 (**i**), HPAC (**j**), SW1990 (**k**), and PANC1 (**l**) after 24 h treatment with MRTX1133. (**m**–**p**). mRNA expression levels of NOXA-coding gene *PMAIP1* in AsPC1 (**m**), HPAC (**n**), SW1990 (**o**), and PANC1 (**p**) after 24 h treatment with MRTX1133. Data in (**i**–**p**) are presented as mean ± SD (n = 3 biological replicates). ** *p* < 0.01, *** *p* < 0.001, **** *p* < 0.0001, ^ns^ *p* > 0.05, i.e., not significant compared to untreated (control) cells as determined by one-way ANOVA and Dunnett’s multiple comparisons test. Since ERK1 and ERK2 have very close molecular weights (~44 and ~42 kDa), the two bands merged and appeared as a single band in panels (**a**–**d**) (and subsequent figures) due to the limited resolution in gradient gels. Original western blots are presented in File S1.

In our previous study, we found that G12Ci sotorasib increases BIM expression in G12C-mutated tumor cell lines [21]. Here, we wondered whether MRTX1133 can also induce BIM expression in G12D-mutated PDAC cells. Consistent with our hypothesis, we found that MRTX1133 can substantially increase BIM expression in multiple G12D-mutated PDAC cell lines, including both p53-mutated (AsPC1, HPAC, and PANC1) and p53 wild-type (SW1990) (Figure 2a–h). However, the increase in BIM levels was cell line-dependent, with the greatest increase in SW1990 cells and the least in PANC1 cells. In addition, the expression of NOXA—a pro-apoptotic protein that endogenously binds to and inhibits anti-apoptotic MCL-1—was substantially reduced after MRTX1133 treatment in all these cell lines, with the greatest reduction in PANC1 cells (Figure 2a–h). On the contrary, MRTX1133 treatment exerted no observable effects on expressions of BCL-xL and MCL-1 (Supplementary Figure S2a–d). Interestingly, SW1990 cells also showed the greatest KRAS inhibition, as indicated by decreased phosphorylated (p)-ERK1/2 and p-Akt compared to other cell lines. The modest increase in BIM in PANC1 cells was correlated with its lesser sensitivity to KRAS inhibition [12,34,34]. In addition, MRTX1133 treatment leads to a compensatory increase in p-AKT, reflecting an activation of the PI3K/AKT pathway, which can provide an alternative route for cell survival and proliferation, potentially reducing the sensitivity of PANC1 cells to MRTX1133. Additionally, minimal inhibition of p-S6 suggests incomplete suppression of mTOR signaling, further contributing to reduced efficacy of MRTX1133 in PANC1 cells. In HPAC cells, p-AKT and p-S6 inhibitions were observed at relatively higher doses of MRTX1133. We confirmed that MRTX1133-mediated BIM upregulation and NOXA downregulation were specific to G12D-mutated PDAC cells, as MRTX1133 exerted no considerable effect on their expressions in KRAS wild-type BxPC3 cells (Supplementary Figure S3a,b). Also, MRTX1133 treatment induced no observable effects on BCL-xL and MCL-1 expressions in BxPC3 cells (Supplementary Figure S3c).

Next, we evaluated whether MRTX1133 could modulate mRNA levels of genes encoding BIM and NOXA in these PDAC cell lines. We found a significant dose-dependent increase in *BCL2L11* (BIM-coding gene) and a reduction in *PMAIP1* (NOXA-coding gene) mRNA levels, in correlation with their corresponding protein levels after MRTX1133 treatment (Figure 2i–p). For example, the upregulation of *BCL2L11* was least, and the downregulation of *PMAIP1* was greatest in PANC1 cells (Figure 2l,p).

### 3.3. DT2216 Releases MRTX1133-Induced BIM from BCL-xL, and Everolimus Restores NOXA That Binds to MCL-1

**Figure 3:**
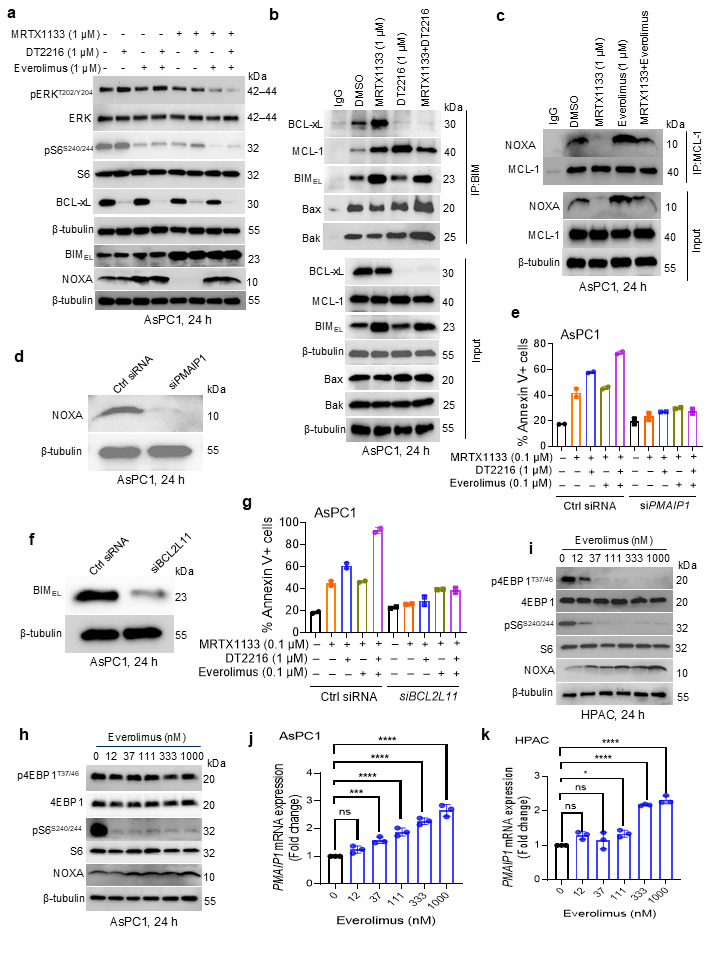
Everolimus-mediated mTOR inhibition increases NOXA and DT2216-mediated BCL-xL degradation releases BIM. (**a**). Immunoblot analyses of phosphorylated (p)- and total- ERK and S6, BCL-xL, BIM, and NOXA in AsPC1 cells after treatment with MRTX1133, DT2216, everolimus, and their combinations as indicated for 24 h. (**b**). Immunoprecipitation analysis of BIM in AsPC1 cells after treatment with DMSO, MRTX1133, DT2216, or MRTX1133+DT2216 for 24 h, and the immunoprecipitated as well as input samples were subjected to immunoblot analysis of BCL-X_L_, MCL-1, and BIM. (**c**). Immunoprecipitation analysis of MCL-1 in AsPC1 cells after treatment with DMSO, MRTX1133, everolimus, or MRTX1133+everolimus for 24 h, and the immunoprecipitated as well as input samples were subjected to immunoblot analysis of NOXA and MCL-1. (**d**,**e**). siRNA-mediated *PMAIP1* (gene encoding NOXA) knockdown in AsPC1 cells as confirmed by immunoblotting (**d**), and percentage annexin V^+^ apoptotic population in Control (Ctrl) siRNA and *PMAIP1* siRNA exposed AsPC1 cells after treatment with MRTX1133 and its combinations with DT2216 and/or everolimus at indicated concentrations for 48 h (**e**). Data in panel (**e**) are presented as mean ± SD (n = 2 independent experiments). (**f**,**g**). siRNA-mediated *BCL2L11* (gene encoding BIM) knockdown in AsPC1 cells, as confirmed by immunoblotting (**f**), and percentage annexin V+ apoptotic population in Control (Ctrl) siRNA and BCL2L11 siRNA-exposed AsPC1 cells after treatment with MRTX1133 and its combinations with DT2216 and/or everolimus at indicated concentrations for 48 h (**g**). Data in panel (**g**) are presented as mean ± SD (n = 2 independent experiments). (**h**,**i**). Immunoblot analyses of phosphorylated (p)- and total- 4EBP1 and S6, and NOXA in AsPC1 (**f**), and HPAC cells (**g**) after treatment with indicated concentrations of everolimus for 24 h. (**j**,**k**). mRNA expression levels of NOXA-coding gene *PMAIP1* in AsPC1 (**h**) and HPAC cells (**i**) after 24 h treatment with MRTX1133. Data are presented as mean ± SD (n = 3 biological replicates). * *p* < 0.05, *** *p* < 0.001, **** *p* < 0.0001, ^ns^ *p* > 0.05, i.e., not significant compared to untreated (control) cells as determined by one-way ANOVA and Dunnett’s multiple comparisons test. β-tubulin was used as an equal loading control in all immunoblots. Original western blots are presented in File S1.

To elucidate the mechanisms by which DT2216 and everolimus potentiate the anti-tumor effect of MRTX1133, we first assessed their effects, as single agents and combinations, on KRAS signaling as well as on BCL-xL, BIM, and NOXA expressions. The triple combination leads to enhanced inhibition of p-ERK and p-S6 coupled with BCL-xL degradation, BIM upregulation, and restoration of NOXA (Figure 3a).

We have recently reported that BCL-xL sequesters G12Ci-induced BIM, thus decreasing pool of free BIM to induce apoptosis in G12C-mutated tumor cells [21]. Here, we wondered whether MRTX1133-induced BIM is also sequestered by BCL-xL, and whether DT2216-mediated BCL-xL degradation can release BIM. Our immunoprecipitation (IP) analysis showed that BCL-xL binds to BIM induced by MRTX1133 in AsPC1 cells. Further treatment with DT2216 led to BCL-xL degradation and BIM release. BIM has a propensity to bind not only with BCL-xL but also with MCL-1; therefore, we immunoblotted IP: BIM samples with an MCL-1 antibody. It turned out that MCL-1 also binds to BIM, though with lesser intensity than BCL-xL. Furthermore, MCL-1 binds to a portion of MRTX1133-induced BIM, and upon DT2216 treatment, slightly more BIM is available to bind MCL-1 because of the unavailability of BCL-xL. However, with the MRTX1133/DT2216 combination treatment, BIM binding to MCL-1 was slightly reduced compared to DT2216 alone, suggesting that additional free BIM is available. To further investigate this, we performed immunoblotting analysis of BAX and BAK in IP: BIM samples. These results show that after combination treatment with MRTX1133+DT2216, increased BIM binds to BAX and BAK (Figure 3b). The BIM binding to BAX/BAK can potentially facilitate their activation to induce mitochondrial apoptosis as has been reported [35,36,37]. In another experiment, we immunoprecipitated MCL-1 and immunoblotted these samples with NOXA antibody to determine whether increased NOXA by everolimus can promote MCL-1/NOXA complex formation. We found that MRTX1133 treatment leads to diminished NOXA/MCL-1 complex formation, while co-treatment with everolimus promotes NOXA/MCL-1 complex formation (Figure 3c). The binding of NOXA to MCL-1 has been shown to inhibit MCL-1 and enables apoptosis induction [38,39]. We extended this mechanistic validation to HPAC (relatively sensitive to MRTX1133) and PANC1 (relatively resistant to MRTX1133) cells. In HPAC cells, the results were similar to those observed in AsPC1 (Supplementary Figure S4a–c). In PANC1 cells, on the other hand, the triple combination leads to inhibition of p-ERK, but stabilization of NOXA was not very obvious. Further, there was only minimal binding of BIM to BCL-xL or MCL-1 in MRTX1133-treated PANC1 cells. Similarly, there was almost negligible binding between NOXA and MCL-1 (Supplementary Figure S4d–f).

To further demonstrate the functional role of NOXA in mediating apoptosis, we performed siRNA-mediated knockdown of *PMAIP1* (the gene encoding NOXA) in AsPC1 cells and assessed apoptosis using Annexin V/PI staining and flow cytometry following treatment with MRTX1133 alone and in combination with everolimus ± DT2216, compared to control siRNA-exposed cells. *PMAIP1* knockdown remarkably diminished apoptosis induction by MRTX1133/everolimus ± DT2216 combinations (Figure 3d,e). These functional data suggest that NOXA induction by everolimus facilitates robust apoptosis induction by the triple combination. Similarly, we knocked down *BCL2L11* (BIM-coding gene) in AsPC1 cells and then evaluated the induction of apoptosis by MRTX1133 alone and in combination with DT2216 and/or everolimus. We found that BIM depletion abrogates the triple combination-mediated induction of apoptosis, indicating that BIM is necessary for the observed pro-apoptotic effect (Figure 3f,g).

Furthermore, we found that everolimus induces a dose-dependent upregulation of NOXA at both the protein and mRNA levels in G12D-mutated AsPC1 and HPAC cell lines (Figure 3h–k). However, unlike our and others’ published reports that mTOR inhibition causes MCL-1 suppression in various tumor cell lines [29,40,41], everolimus did not alter the expression of MCL-1 in the G12D-mutated PDAC cell lines (Supplementary Figure S4g–i). These results suggest that a combination of DT2216 and everolimus can enhance the anti-tumor activity of MRTX1133 associated largely with BIM release and NOXA-mediated MCL-1 inhibition, respectively, and, in part, with ERK and mTOR inhibition. Overall, the efficacy of the triple combination is associated with the concurrent enhancement of apoptosis and suppression of compensatory survival signaling.

We also evaluated the effect of direct targeting of MCL-1 with the selective inhibitor S63845 in G12D-mutated AsPC1 cells, both as a single agent and in combination with MRTX1133 and/or DT2216. These results indicate that the combination of S63845 with MRTX1133 significantly increased cell loss compared to MRTX1133 alone, and the triple combination of MRTX1133, S63845, and DT2216 was significantly more potent than single agents and two-drug combinations (Supplementary Figure S5a–c).

### 3.4. The DT2216/Everolimus Combination Enhances the Efficacy of MRTX1133 In Vivo

**Figure 4:**
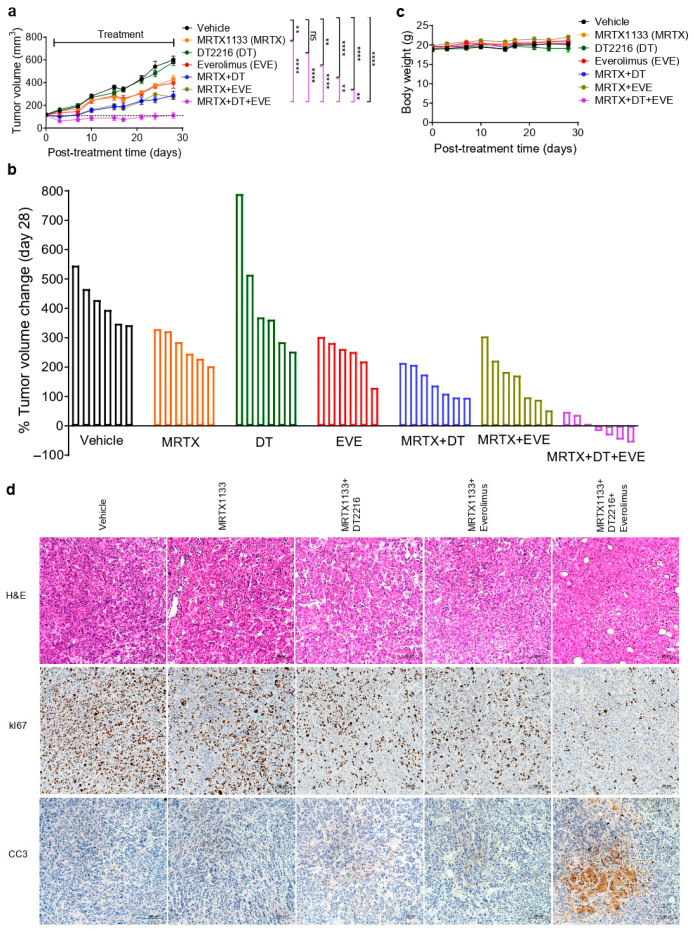
The DT2216/everolimus combination enhances the efficacy of MRTX1133 in AsPC1 xenograft model. (**a**). Tumor volume changes in AsPC1 xenografts after they were treated with vehicle, MRTX1133 (MRTX, 3 mg/kg, b.i.d, 5 days ON/2 days OFF, i.p.), DT2216 (DT, 15 mg/kg, 2×/week, i.p.), everolimus (EVE, 2.5 mg/kg, 2×/week, p.o.) or their combinations as indicated. Data are presented as mean ± SEM (n = 6 mice per group in vehicle and single treatment arms, n = 7 mice per group in combination treatment arms). Statistical significance was determined by a two-sided Student’s *t*-test at the last tumor measurement, i.e., day 28. ** *p* < 0.01, **** *p* < 0.0001, ^ns^ *p* > 0.05 i.e., not significant. (**b**). Percent changes in tumor volumes of individual mice at the end of treatment as compared to their baseline tumor volumes. (**c**). Mouse body weight changes after treatment, as in (**a**). (**d**), H&E, Ki67, and CC3 staining in tumor samples from PD study (n = 3 mice per group). Images were captured under 10× magnification.

We evaluated the antitumor efficacy of the combination of MRTX1133 with DT2216/everolimus in vivo using the AsPC1 xenograft model. As anticipated, DT2216 alone had no significant effect on tumor growth. MRTX1133 and everolimus alone show minimal tumor growth inhibition, whereas the combination of DT2216 or everolimus with MRTX1133 yields significant tumor growth inhibition compared to the individual agents. Importantly, the triple combination leads to the greatest tumor inhibition, which was significant compared to all other groups. More importantly, the triple combination caused tumor regressions in five out of seven mice in the group (Figure 4a,b). Neither the two-drug combinations nor the triple combination leads to any changes in mouse body weights (Figure 4c). In addition, the triple combination neither showed any further reduction in blood cell counts (platelets, WBCs and RBCs) compared to single agents (Supplementary Figure S6a–c), nor showed any significant increases in ALT/AST enzyme activities, which are indicative of normal liver function (Supplementary Figure S6d,e). Of note, DT2216 alone reduced platelet levels, but the levels remained above 0.1 million per µL of blood, which is considered clinically safe [21,27,29,42]. Moreover, the addition of everolimus and/or MRTX1133 did not cause further reduction in platelet counts. These results suggest that the triple combination of MRTX1133 with DT2216 and everolimus is more efficacious than the two-drug combinations without causing apparent toxicity in mice under the assessed parameters (mouse body weights, blood cell counts, AST/ALT activities).

We next measured PD markers of MRTX1133, DT2216, and everolimus after short-term treatment of AsPC1 xenografts in a separate experiment. DT2216 leads to a substantial reduction in BCL-xL levels, which is sustained in two-drug and triple combination groups. MRTX1133 at a 3 mg/kg b.i.d. dose leads to a substantial reduction of p-ERK, but not of p-S6 and p-AKT. While S6 phosphorylation was effectively reduced after everolimus treatment and sustained in combination-treated tumors. We did not see any further inhibition of p-ERK or p-S6 over that of MRTX1133 or everolimus, respectively, in the combination groups (Supplementary Figure S7a,b). This suggests the triple combination exerts enhanced antitumor efficacy by simultaneously targeting KRAS, BCL-xL, and mTOR. H&E staining of tumor tissues showed that the vehicle group exhibited large, densely packed tumor cells with areas of necrosis. Treatment with MRTX1133 alone moderately reduced tumor size and cellularity, but tumor cells remained substantial. The combination of MRTX1133 with DT2216 or everolimus showed further tumor shrinkage and increased apoptosis. The triple combination of MRTX1133 with DT2216 and everolimus was the most effective, resulting in the smallest tumors with minimal tumor cellularity and increased necrosis, indicating an enhanced antitumor effect (Figure 4d). IHC analysis of Ki-67 expression demonstrated high levels of staining in the vehicle group. Treatment with MRTX1133 moderately reduced Ki67+ cells, and this reduction was enhanced when combined with DT2216 or everolimus. Importantly, the triple combination leads to the greatest reduction in Ki67^+^ cells. On the other hand, increased CC3 staining was observed in tumors from the triple combination group compared with MRTX1133 alone or two-drug combinations (Figure 4d). These results further support that the triple combination leads to enhanced anti-tumor activity by simultaneous inhibition of cell proliferation and induction of apoptosis.

### 3.5. The DT2216/Everolimus Combination Sensitizes Acquired MRTX1133-Resistant AsPC1 Cells In Vitro and in Xenograft Model

**Figure 5:**
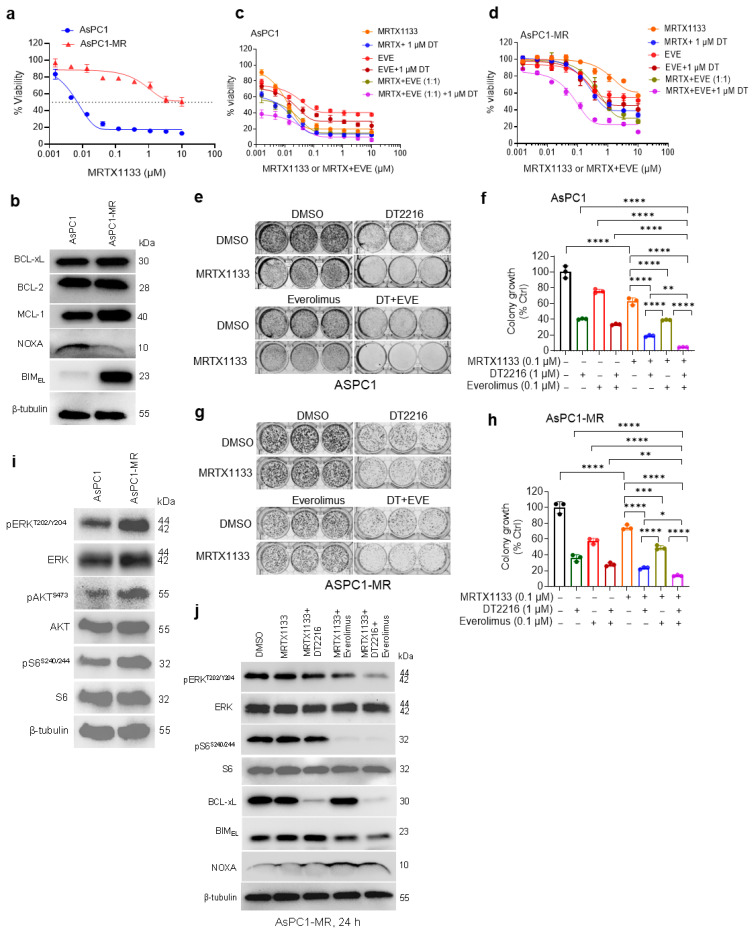
The DT2216/everolimus combination sensitizes resistant cells to MRTX1133 treatment in vitro. (**a**). Viability of MRTX1133-resistant AsPC1 (AsPC1-MR) cells compared to parental AsPC1 cells after treatment with increasing concentrations of MRTX1133 for 72 h. (**b**). Immunoblot analyses of BCL-xL, BCL-2, MCL-1, NOXA, and BIM in AsPC1 and AsPC1-MR cells. (**c**,**d**). Viability of parental AsPC1 (**c**) and AsPC1-MR cells (**d**) after treatment with increasing concentrations of MRTX1133, everolimus (EVE) alone and/or with 1 µM of DT2216 (DT) and/or equimolar (1:1) ratio of everolimus for 72 h. Data are presented as ± SD (n = 3 replicate cell cultures). First points in MRTX + 1 µM DT2216, EVE + 1 µM DT2216 and MRTX + EVE (1:1) + 1 µM DT2216 curves represent 1 µM DT2216 alone. (**e**,**g**). Cell colony images of parental AsPC1 (**e**) and AsPC1-MR (**g**) after treatment with MRTX1133, DT2216, or EVE alone or with dual or triple combinations as indicated for 14 days, followed by crystal violet staining. (**f**,**h**). Colorimetric measurement of colony growth in parental AsPC1 (**f**) and AsPC1-MR cells (**h**). Data in (**a**,**c**,**d**,**f**,**h**) are presented as mean ± SD (n = 3 cell culture replicates). Statistical significance for data in panels (**f**,**h**) was determined by one-way ANOVA and Tukey’s multiple comparisons test, where * *p* < 0.05, ** *p* < 0.01, *** *p* < 0.001, **** *p* < 0.0001. (**i**). Immunoblot analyses of phosphorylated- and total- ERK, AKT, and S6 in AsPC1 and AsPC1-MR cells. (**j**). Immunoblot analyses of phosphorylated- and total- ERK, S6, and AKT, BCL-xL, BIM, and NOXA in AsPC1-MR cells after treatment with MRTX1133 alone or in combination with DT2216 and/or everolimus as indicated. β-tubulin was used as an equal loading control in all immunoblots. Original western blots are presented in File S1.

Acquired resistance is a key factor contributing to the limited clinical efficacy of KRASi. We generated MRTX1133-resistant AsPC1 cells (designated AsPC1-MR) by exposing them to progressively increasing concentrations of MRTX1133. The AsPC1-MR cells showed substantial resistance to MRTX1133 compared with parental AsPC1 cells (Figure 5a). Subsequently, we performed immunoblotting on AsPC1-MR cells and their parental AsPC1 counterparts to assess the expression of selected BCL-2 family proteins. While no noticeable changes were observed in BCL-xL and BCL-2 levels, a substantial increase in BIM, along with a decrease in NOXA, was detected in AsPC1-MR cells compared to parental cells. In addition, AsPC1-MR cells also showed a marked increase in MCL-1 levels (Figure 5b). Next, we investigated whether the combination of DT2216 and everolimus could sensitize AsPC1-MR cells to MRTX1133 treatment. While DT2216 or everolimus alone moderately sensitized AsPC1-MR cells to MRTX1133, the DT2216+everolimus combination markedly enhanced their sensitivity in both short-term cell viability and long-term colony formation assays (Figure 5c–h). Of note, the acquired resistance to MRTX1133 was associated with increased sensitivity to everolimus, suggesting that cellular dependence on the mTOR pathway increases upon development of resistance to MRTX1133, consistent with a previous report [12] (Figure 5c–h). This suggests that mTOR plays a more critical role in the acquired resistance of AsPC1-MR cells to MRTX1133, whereas BCL-xL contributes to intrinsic resistance. Indeed, AsPC1-MR cells exhibited a marked increase in mTOR activation, as evidenced by elevated p-S6 levels. Additionally, increased phosphorylation of ERK and AKT was also observed in AsPC1-MR cells (Figure 5i). Furthermore, we explored the mechanism underlying the sensitizing effect of the DT2216+everolimus combination and found that the combination simultaneously inactivates p-ERK and p-S6, degrades BCL-xL, and restores NOXA expression in AsPC1-MR cells (Figure 5j). Interestingly, everolimus was found to slightly decrease BIM expression in AsPC1-MR cells; however, the remaining BIM levels appeared sufficient to induce cell killing.

**Figure 6:**
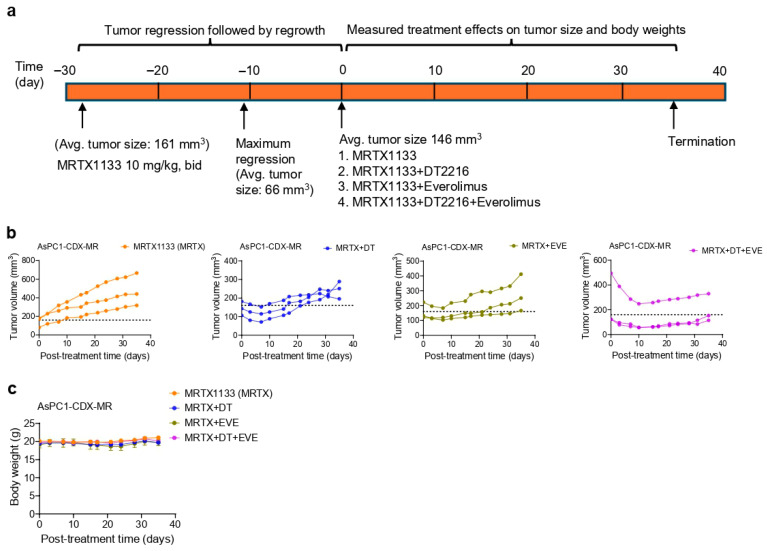
The DT2216/everolimus combination sensitizes resistant AsPC1 xenografts to MRTX1133 treatment. (**a**). Schematic of AsPC1-CDX-MR study design. (**b**). Tumor volume changes in individual AsPC1-CDX-MR mice after they were treated with MRTX1133 (MRTX, 10 mg/kg, b.i.d, i.p.) alone or in combination with DT2216 (DT, 15 mg/kg, 2×/week, i.p.) and/or everolimus (EVE, 2.5 mg/kg, 2×/week, p.o.). (n = 3 mice per group) (**c**). Mouse body weight changes during the course of treatment, as in (**b**).

We next generated an AsPC1-CDX-MR model by treating AsPC1 tumor-bearing mice (average tumor size 161 mm^3^) with MRTX1133 at 10 mg/kg b.i.d. Initially, mice responded to treatment, with continued tumor regression, and the average tumor size reached 66 mm^3^ after 17 days of treatment. Then tumors regrew and reached 146 mm^3^ after 28 days. At this point, tumors were considered resistant in our study. Thereafter, mice were randomized into four groups and treated with MRTX1133 alone, MRTX1133 + DT2216, MRTX1133 + everolimus, or MRTX1133 + DT2216 + everolimus (Figure 6a). As expected, MRTX1133-treated mice exhibited continuous tumor growth. MRTX1133 + DT2216-treated mice exhibited tumor regression for one week before tumors began to regrow. MRTX1133 + everolimus-treated mice showed tumor inhibition for approximately 2 weeks, after which tumors resumed growth. MRTX1133 + DT2216 + everolimus-treated mice demonstrated sustained tumor regression for up to four weeks before tumors began to regrow, albeit more slowly than in the other treatment groups. Notably, one mouse in the MRTX1133 + DT2216 + everolimus group had a significantly larger tumor than the other mice; however, this tumor showed substantial regression following the triple combination treatment (Figure 6b). None of the treatment groups showed changes in body weight during or at the end of the treatment (Figure 6c). The tumor growth inhibition in these mice was associated with BCL-xL degradation by DT2216, reduction of p-ERK by MRTX1133+everolimus, and reduction of p-S6 by everolimus in resistant tumors. Moreover, the triple combination led to enhanced inhibition of p-ERK and p-S6 (Supplementary Figure S8a,b).

## 4. Discussion

Based on our previous study and that of others, G12Ci has been shown to induce minimal apoptosis due to dysregulated expression of BCL-2 family proteins, contributing to both intrinsic and acquired resistance [21,22]. In the current study, we primarily focus on intrinsic resistance mechanisms to G12Di MRTX1133 within G12D-mutant PDAC tumors. Impaired apoptosis (e.g., due to overexpression of anti-apoptotic proteins such as BCL-xL and MCL-1) appears to be a major barrier to MRTX1133′s efficacy, along with activation of compensatory survival pathways, as previously reported [12,13,14,43]. We observed that MRTX1133 can only partially inhibit the colony-forming capabilities of multiple G12D-mutated tumor cell lines with mild to modest apoptosis induction. The triple combination of DT2216 and everolimus with MRTX1133 was found to dramatically eliminate colony growth and was significantly more potent than the two-drug combinations of DT2216/MRTX1133 or everolimus/MRTX1133. In PANC1 cells, the triple combination was significantly more potent than the MRTX1133 or two-drug combinations; however, it could not completely eliminate clonogenic growth, suggesting that PANC1 cells are outliers among G12D-mutated tumor cells. Of note, the two-drug combinations of DT2216/MRTX1133 and everolimus/MRTX1133 exhibited heterogeneous effects across tumor cell lines. The DT2216/MRTX1133 combination was more potent in AsPC1 and SW1990 cells, whereas everolimus/MRTX1133 was more potent in PANC1 cells, and they were similar in HPAC cells. MRTX1133 alone could only induce mild-to-moderate apoptosis; its combination with DT2216 or DT2216/everolimus robustly induced apoptosis. These results confirm our and others’ previous findings that KRAS inhibitors generally act as cytostatic agents, with minimal apoptosis induction as single agents, and therefore require combinations with apoptosis inducers for optimal therapeutic efficacy [21,22]. This is because the impaired expression of anti-apoptotic proteins, particularly BCL-xL and MCL-1, in PDAC cells limits the ability of MRTX1133 monotherapy to induce cell death, even when KRAS signaling is effectively suppressed [21,31,44,45,46].

Furthermore, we demonstrated that MRTX1133 abolishes KRAS signaling, predominantly through inhibition of the ERK/MAPK pathway, specifically in G12D-mutated PDAC cell lines. Notably, the PI3K/Akt/mTOR pathway was differentially inhibited by MRTX1133 across G12D-mutated PDAC cell lines, whereas it was not significantly inhibited in PANC1 cells, which have previously been reported to be relatively resistant to KRAS signaling inhibitors [12,34,34]. Since KRAS regulates both ERK/MAPK and PI3K/Akt pathways, both p-ERK and p-Akt are regarded as biomarkers of KRAS inhibition [4,6]. However, our data indicate that MRTX1133-responsive cells (AsPC1, HPAC, and SW1990) exhibit more pronounced decreases in p-Akt compared to resistant cells (PANC1), which maintain PI3K/Akt signaling despite KRAS inhibition. Therefore, our data suggest that p-Akt inhibition is a better predictor of response to MRTX1133. Importantly, we observed significant upregulation of BIM and downregulation of NOXA following MRTX1133 treatment in G12D-mutated PDAC cell lines, whereas expression of BCL-xL and MCL-1 remained unaffected.

Apoptosis is regulated by the balance between pro-apoptotic proteins (such as BIM and NOXA) and anti-apoptotic proteins (such as BCL-xL and MCL-1) [36,37]. By modulating the expression of BIM and NOXA, MRTX1133 can induce a dependence on BCL-xL and MCL-1 in G12D-mutated PDAC cells. BCL-xL and MCL-1 are overexpressed and play a critical role in promoting survival in many solid tumors, including PDAC [44,45,46]. The increased levels of BIM can be sequestered by high amounts of BCL-xL, preventing BIM from activating BAK/BAX, an essential step for intrinsic or mitochondrial-mediated apoptosis [36,37,44]. Additionally, the reduced expression of NOXA may unleash MCL-1, which further binds to BIM, contributing to a survival advantage for tumor cells [36]. Since NOXA is a pro-apoptotic BH3-only protein that endogenously binds to and inhibits MCL-1 [35,47,48], the reduction of NOXA by MRTX1133 can potentially contribute to apoptosis resistance through an increased activity of MCL-1. Therefore, strategies to restore NOXA expression may enhance the efficacy of MRTX1133 by promoting apoptosis. Based on this, we hypothesize that BCL-xL degradation, combined with MCL-1 inhibition via NOXA restoration, could be a potential mechanism for the enhanced efficacy of MRTX1133. BCL-xL degradation can be achieved with the PROTAC DT2216 without affecting platelets, unlike conventional BCL-xL inhibitors [27]. However, available MCL-1 inhibitors lack tumor selectivity and can induce cardiotoxicity and hepatotoxicity, particularly when combined with BCL-xL inhibitors or degraders [49,50]. Therefore, MCL-1 inhibition via NOXA could be a clinically relevant strategy. The mTOR inhibitors have been shown to suppress MCL-1 expression in various tumor cells, as demonstrated by us and others [29,40,41]. To our surprise, the mTOR inhibitor everolimus did not reduce MCL-1 levels in G12D-mutated PDAC cells, but it notably upregulated NOXA expression. This could be partly because mTOR inhibition is known to trigger a variety of cellular stress responses, including inhibition of cap-dependent translation and induction of the integrated stress response (ISR) [51,52]. It has been reported that under certain stress conditions, NOXA can be upregulated via ATF4, a key transcription factor activated during ISR. Therefore, mTOR inhibition may increase NOXA expression by activating the ATF4 pathway, leading to transcriptional upregulation of *PMAIP1*, the gene encoding NOXA [53,54]. Further, Immunoprecipitation analysis revealed that the increased NOXA following everolimus treatment binds to MCL-1, potentially inhibiting its function. However, everolimus can enhance apoptosis not only by partially restoring NOXA but also by inhibiting mTOR signaling, because feedback activation of mTOR signaling is known to contribute to resistance to KRAS inhibitors [13,19]. In addition, we demonstrated NOXA’s requirement in apoptosis induction by MRTX1133/everolimus ± DT2216 combinations using genetic knockdown of *PMAIP1* (the gene encoding NOXA) in AsPC1 cells. Additionally, DT2216 induced BCL-xL degradation, which is expected to release bound BIM and increase the pool of BIM available to engage pro-apoptotic effectors.

In vivo, we found that MRTX1133 alone or in combination with DT2216 or everolimus can slow tumor growth. However, the triple combination not only completely inhibited tumor growth but also induced tumor regressions in some mice. Moreover, we did not observe any significant changes in mouse body weights, increased toxicity to blood cells, or AST/ALT activities, suggesting that the triple combination is apparently tolerable in mice under the assessed parameters. Notably, DT2216, as a single agent, had no significant antitumor effect despite BCL-xL degradation and inhibition of colony growth in vitro. The lack of single-agent efficacy of DT2216 in vivo is likely due to compensatory survival signals in the tumor microenvironment, and/or a shift in reliance on other anti-apoptotic proteins, such as MCL-1, upon BCL-xL depletion [32,55,56,57,58]. Therefore, loss of BCL-xL alone is insufficient to exert an anti-tumor effect unless accompanied by pro-apoptotic signals, such as BIM upregulation, which is achieved with MRTX1133 co-treatment.

Through PD analysis, we found that MRTX1133, DT2216, and everolimus inhibit or degrade their respective targets, suggesting on-target antitumor activity. In addition, the triple combination leads to more pronounced inhibition of Ki67 and induction of cleaved caspase-3 in tumors after short-term treatment. Therefore, the tumor-regressive activity of the triple combination was likely due to simultaneous inhibition of tumor cell proliferation and enhanced apoptosis. However, we did not observe enhanced inhibition of p-ERK or p-S6 in tumors with the triple combination, unlike what was observed in vitro, which might be because these mice were treated for only 5 days.

We next established acquired MRTX1133 -resistant AsPC1 cells (AsPC1-MR) and a xenograft model. In AsPC1-MR cells, we found decreased NOXA expression and increased BIM expression compared to parental AsPC1 cells. In addition, these cells showed increased activation of ERK, AKT, and mTOR. The triple combination of MRTX1133, DT2216, and everolimus significantly inhibited colony growth compared to MRTX1133 alone or two-drug combinations in AsPC1-MR cells. Interestingly, everolimus was slightly more effective in AsPC1-MR cells than in parental AsPC1 cells. This might have resulted from increased dependence of AsPC1-MR cells on the mTOR pathway, as indicated by higher mTOR activity, compared to parental AsPC1 cells, consistent with a published report [12]. We also found increased ERK inhibition following triple-combination treatment in AsPC1-MR cells, suggesting that ERK activity was suppressed. In the AsPC1 xenograft model that became resistant after continuous treatment with MRTX1133, the triple combination leads to sustained tumor regressions for a longer duration than two-drug combinations. However, we hypothesize that MRTX1133-resistant cells harboring secondary KRAS mutations—particularly those that directly affect MRTX1133 binding—are unlikely to respond to the combination of everolimus and DT2216, as MRTX1133 would no longer be effective as a backbone agent. Our combination approach is intended to enhance the apoptotic response in MRTX1133-sensitive or partially resistant cells, but it is unlikely to overcome resistance driven by secondary KRAS mutations. However, this combination strategy may delay or reduce the emergence of acquired resistance by inducing robust apoptosis and preventing the survival of residual tumor cells.

In addition to apoptotic dysregulation, EMT has been reported as a key driver of resistance to KRASi in PDAC, particularly in PANC1 cells, which exhibit a mesenchymal phenotype associated with reduced drug sensitivity. EMT-mediated plasticity enables tumor cells to evade KRAS-targeted therapy by activating alternative survival pathways and promoting invasive characteristics [12,13,14]. While our study did not directly assess EMT, the limited response observed in PANC1 cells may partly reflect EMT-related resistance, underscoring the importance of future investigations into strategies that combine KRASi with agents targeting EMT or its downstream signaling. Accordingly, our study does not imply uniform efficacy across all G12D-mutated PDAC lines and indicates that more epithelial-like models respond most robustly to this regimen.

For our studies, we used 3 mg/kg b.i.d. and 10 mg/kg b.i.d. dosing of MRTX1133 for AsPC1 xenograft and acquired resistant AsPC1 xenograft, respectively, which are significantly lower than maximum tolerated dose (MTD) of 30 mg/kg b.i.d. For the resistance model (Figure 6), we increased the dose of MRTX1133 to 10 mg/kg b.i.d. dosing based on results at 3 mg/kg b.i.d. dosing (Figure 4) that did not induce tumor regression as a single agent and tumors continued to grow from the outset. Since our goal in the resistance model was to first achieve tumor regression followed by regrowth, we selected the higher dose. These combinations were tolerated in mice, with no overt toxicity under the assessed parameters (mouse body weights, blood cell counts, and AST/ALT enzymatic activities). Since the higher doses of MRTX1133 (up to 30 mg/kg, i.e., MTD) have been used in reported combination studies [4,59,60], future studies will evaluate whether increasing the dose of MRTX1133 can lead to complete responses in patient-derived xenograft models of G12D-mutated PDAC without increasing normal toxicities.

## 5. Limitations of the Study

While our mechanistic model is supported by immunoblotting, immunoprecipitation, and correlative signaling analyses, the proposed changes in specific protein–protein interactions and the pathway responsible for NOXA upregulation remain inferential. The protein–protein interaction data in this study were obtained using one-directional immunoprecipitation assays, and reciprocal IPs or orthogonal assays such as Proximity Ligation Assay (PLA) or mitochondrial fractionation (cytochrome c release) were not performed.

Since desmoplastic stroma may be an important resistance mechanism to MRTX1133 in PDAC patients, our study has a limitation: it does not use immunocompetent mouse models (such as orthotopic syngeneic models and genetically engineered mouse models (GEMMs)) to more closely recapitulate human disease. In addition, for our acquired resistance model, we used three mice per group to assess resistance and sensitization to MRTX1133. Given the smaller cohort of mice used, this pilot study requires further validation with larger cohorts and alternative models (e.g., transplantation of resistant tumors into new mice) to more rigorously investigate the combination effect on acquired MRTX1133 resistance. Also, we have not investigated whether the triple combination can enhance cell death susceptibility and antitumor efficacy in tumor cell lines harboring various types of secondary KRAS mutations.

## 6. Conclusions

Our study provides strong evidence that the combination of DT2216 and everolimus enhances the anti-tumor efficacy of MRTX1133. Using in vitro and in vivo models, we demonstrated that the triple combination exhibited significantly enhanced anti-clonogenic and tumor-inhibitory activities compared with MRTX1133 alone, and this was associated with increased apoptotic cell death. These findings highlight the potential of this combination therapy in overcoming resistance mechanisms in G12D-mutated PDAC. After further evaluation in PDX models and spontaneous tumor models (such as the KPC model) of G12D-mutated PDAC, this approach could pave the way for future clinical exploration of this strategy to improve G12Di efficacy in patients.

## Data Availability

All the data related to this manuscript are available in the main figures, supplemental information, or upon reasonable request from the corresponding authors.

## Supplementary Materials

The following supporting information can be downloaded at: https://www.mdpi.com/article/10.3390/cancers18060920/s1, **Figure S1**. (**a–c**). Immunoblot analyses of BCL-xL, cleaved caspase-3 (CC3) and cleaved PARP in AsPC1 (a), HPAC (b) and SW1990 cells (c) after they were treated with MRTX1133, DT2216, everolimus and their combinations as indicated for 48 h. β-tubulin was used as an equal loading control in all immunoblots. (**d**). Percentage annexin V+ apoptotic population without and with 10 µM QVD-pretreated AsPC1 cells after treatment with MRTX1133 and its combinations with DT2216 and/or everolimus at indicated concentrations for 48 h. **Figure S2**. (**a**–**d**). Immunoblot analyses of BCL-xL and MCL-1 in AsPC1 (a), HPAC (b), SW1990 (c), and PANC1 (d) cells after they were treated with indicated concentrations of MRTX1133 for 24 h. The β-tubulin was used as an equal loading control. **Figure S3**. (**a**). Immunoblot analyses of phosphorylated- and total- ERK, AKT and S6, BIM extra-long isoform (BIM_EL_) and NOXA in AsPC1 cells after they were treated with indicated concentrations of MRTX1133 for 24 h. (**b**). Densitometric analysis of BIM_EL_ and NOXA immunoblots in AsPC1 cells. (**c**). Immunoblot analyses of BCL-xL and MCL-1 in BxPC3 cells. The β-tubulin was used as an equal loading control. **Figure S4**. (**a**–**c**). (**a**,**d**). Immunoblot analyses of phosphorylated- and total- ERK and S6, BCL-xL, BIM and NOXA in HPAC (a) and PANC1 (d) cells after they were treated with MRTX1133, DT2216, everolimus and their combinations as indicated for 24 h. (**b**,**e**). Immunoprecipitation analysis of BIM in HPAC (b) and PANC1 (e) cells after they were treated with DMSO, MRTX1133, DT2216 or MRTX1133+DT2216 for 24 h, and the immunoprecipitated as well as input samples were subjected to immunoblot analysis of BCL-X_L_, MCL-1, and BIM. (**c**,**f**). Immunoprecipitation analysis of MCL-1 in HPAC (c) and PANC1 (f) cells after they were treated with DMSO, MRTX1133, everolimus or MRTX1133+everolimus for 24 h, and the immunoprecipitated as well as input samples were subjected to immunoblot analysis of NOXA and MCL-1. (**g**–**i**). Immunoblot analyses of MCL-1 in AsPC1 (g), HPAC (h) and SW1990 cells (i) after they were treated with indicated concentrations of everolimus for 24 h. The β-tubulin was used as an equal loading control. **Figure S5**. (**a**). Viability of parental AsPC1 cells after they were treated with increasing concentrations of MRTX1133, S63845 alone and/or with 1 µM of DT2216 and/or equimolar (1:1) ratio of S63845 for 72 h. Data are presented as ± SD (n= 3 replicate cell cultures). First points in MRTX + 1 µM DT2216, S63845 + 1 µM DT2216 and MRTX + S63845 (1:1) + 1 µM DT2216 represent 1 µM DT2216 alone. (**b**). Colony images of AsPC1 cells after they were treated with MRTX1133, DT2216, S63845 and their combinations as indicated for 14 days followed by crystal violet staining. (**c**). Colorimetric measurement of colony growth in AsPC1 cells. Data are presented as mean ± SD (n = 3 cell culture replicates). Statistical significance was determined by one-way ANOVA and Tukey’s multiple comparisons test, where *****p* <0.0001. **Figure S6**. (a–c). Enumeration of platelets (a), WBCs (b), and RBCs (c) 24 h after the final dose of DT2216. Data are presented as mean ± SEM (n = 6–7 mice per group). (**d**,**e**). ALT (d) and AST (e) activities in mouse serum as measured 24 h after final dose of DT2216. Data are presented as mean ± SEM (n = 3 mice per group). **Figure S7.** The MRTX1133, DT2216 and everolimus demonstrate their respective target inhibition in AsPC1 xenografts. (**a**). Immunoblot analyses of BCL-xL, phosphorylated- and total- ERK, S6, and AKT in AsPC1 xenograft mice after they were treated with vehicle, MRTX1133 (MRTX, 3 mg/kg, b.i.d, Day 1–5, i.p.), DT2216 (DT, 15 mg/kg, Day 1 and Day 5, i.p.), everolimus (EVE, 2.5 mg/kg, Day 1 and Day 5, p.o.) or their combinations as indicated and tumors were harvested 24 h after 2^nd^ dose of DT2216 i.e., Day 6. The β-tubulin was used as an equal loading control. (**b**). Densitometric analysis of immunoblots as in (**a**). Data are presented as mean ± SEM (n = 3 mice per group). **Figure S8**. (**a**). Immunoblot analyses of phosphorylated- and total- ERK and S6, BCL-xL, BIM and NOXA in AsPC1-CDX-MR tumors after indicated treatments. (**b**). Densitometric analysis of immunoblots as in (**a**). Data are presented as mean ± SEM (n = 3 mice per group). **Table S1**. Antibodies used in immunoblotting. File S1: Original western blots.
