# Supplementary material for: The Combination of a BCL-xL PROTAC and an mTOR Inhibitor Sensitizes Pancreatic Ductal Adenocarcinoma to KRASG12D Inhibitor Treatment
Source: Cancers (Basel). 2026 Mar 12;18(6):920. doi: 10.3390/cancers18060920 (PMC13025216; doi:10.3390/cancers18060920)

Figure 2

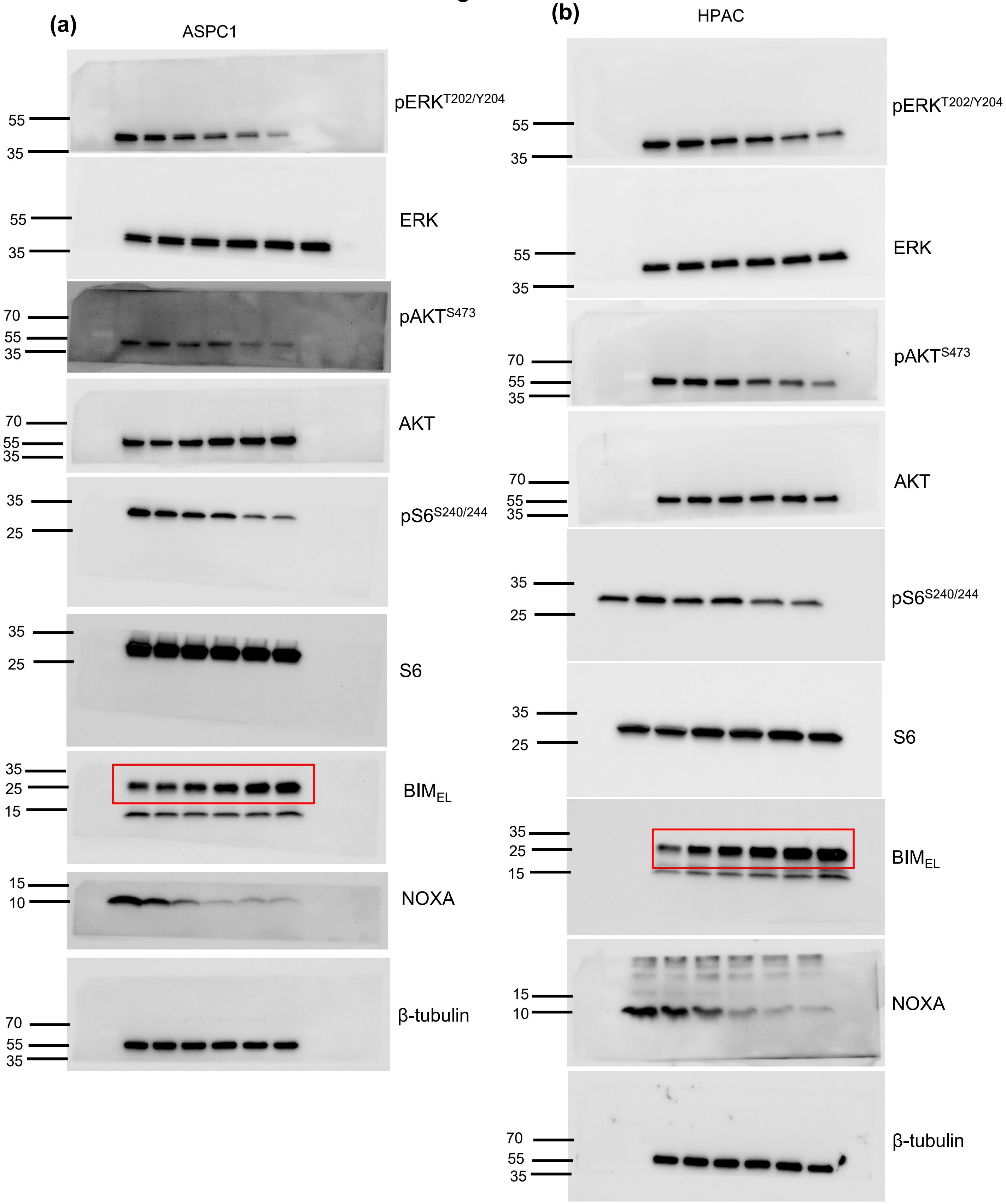
(a)
ASPC1
pERK T202/Y204
ERK
pAKT S473
AKT
pS6 S240/244
S6
BIM EL
NOXA
β-tubulin
55
35
70
25
15
10
(b)
HPAC
pERK T202/Y204
ERK
pAKT S473
AKT
pS6 S240/244
S6
BIM EL
NOXA
β-tubulin

Figure 2

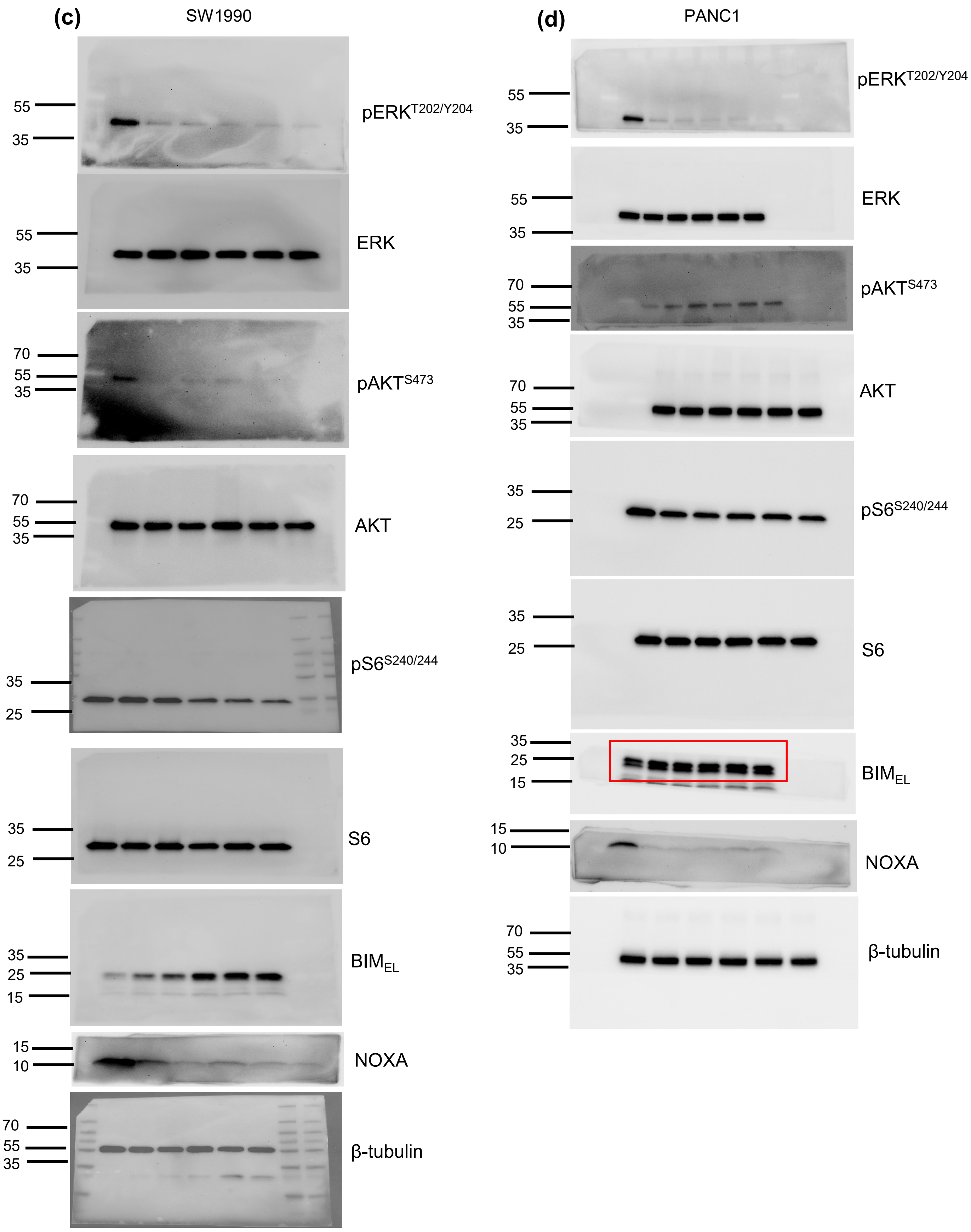

(c)
SW1990
(d)
PANC1
55
35
pERK T202/Y204
ERK
70
pAKT S473
AKT
25
pS6 S240/244
S6
15
BIM EL
10
NOXA
β-tubulin

Figure 3

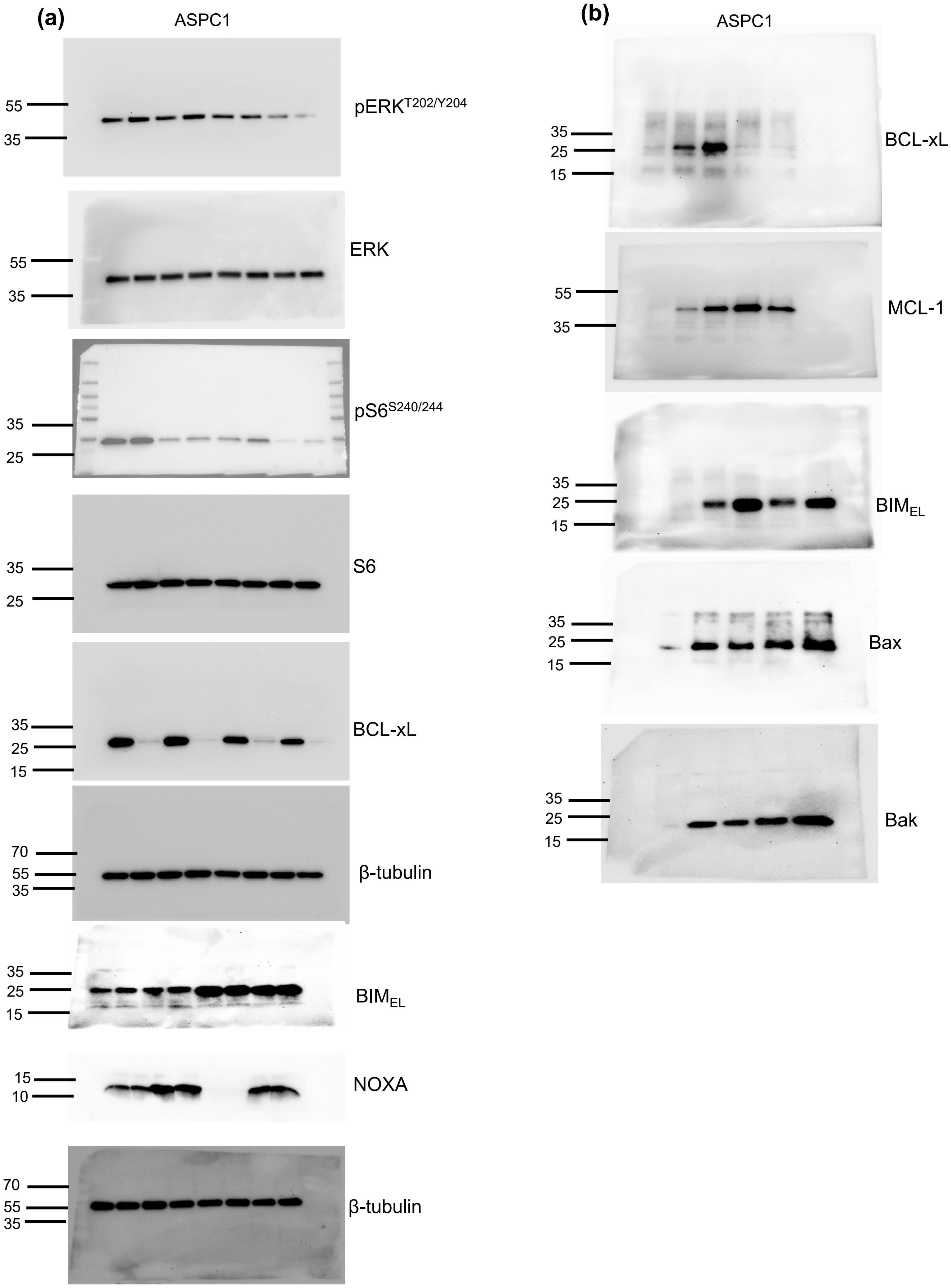
(a)
ASPC1
55
35
pERK T202/Y204
55
35
ERK
35
25
pS6 S240/244
35
25
S6
35
25
15
BCL-xL
70
55
35
β-tubulin
35
25
15
BIM EL
15
10
NOXA
70
55
35
β-tubulin
(b)
ASPC1
35
25
15
BCL-xL
55
35
MCL-1
35
25
15
BIM EL
35
25
15
Bax
35
25
15
Bak

Figure 3

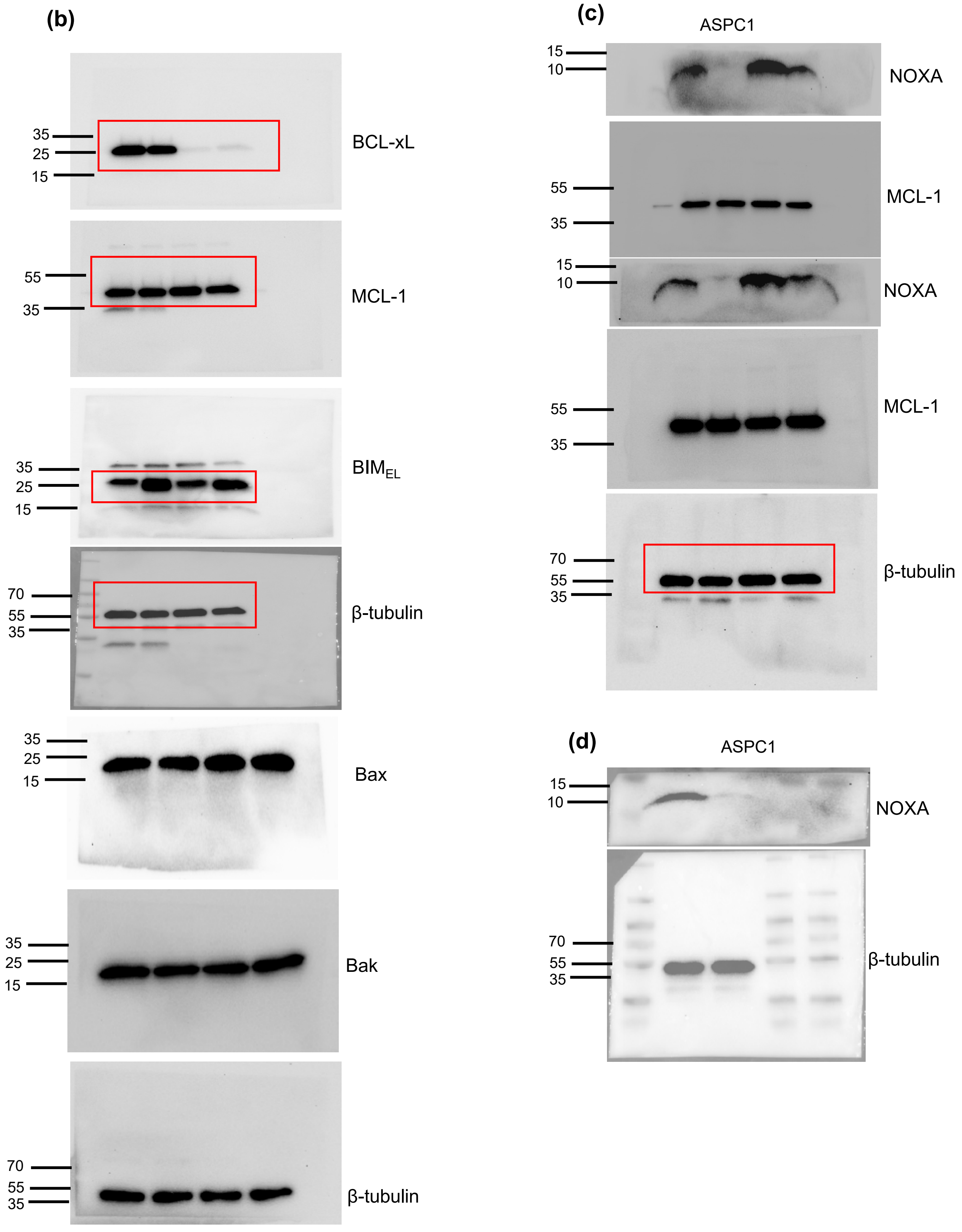
(b)
BCL-xL
MCL-1
BIMEL
β-tubulin
Bax
Bak
β-tubulin
35
25
15
55
70
(c)
ASPC1
NOXA
MCL-1
NOXA
MCL-1
β-tubulin
15
10
55
35
70
(d)
ASPC1
NOXA
β-tubulin

Figure 3

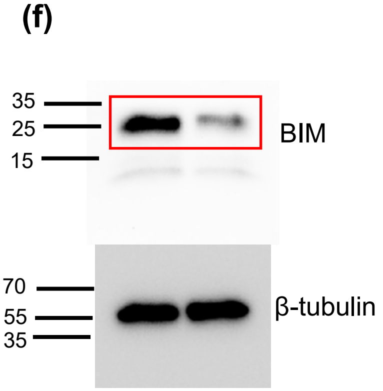
(f)
35
25
15
BIM
70
55
35
β-tubulin

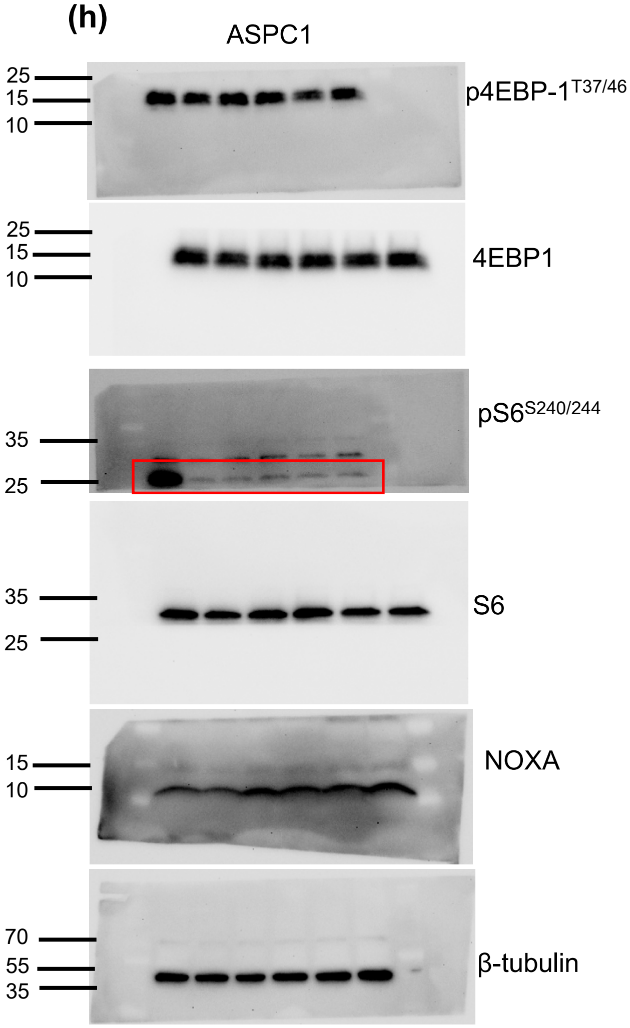
(h)
ASPC1
25
15
10
p4EBP-1T37/46
25
15
10
4EBP1
35
25
pS6S240/244
35
25
S6
15
10
NOXA
70
55
35
β-tubulin

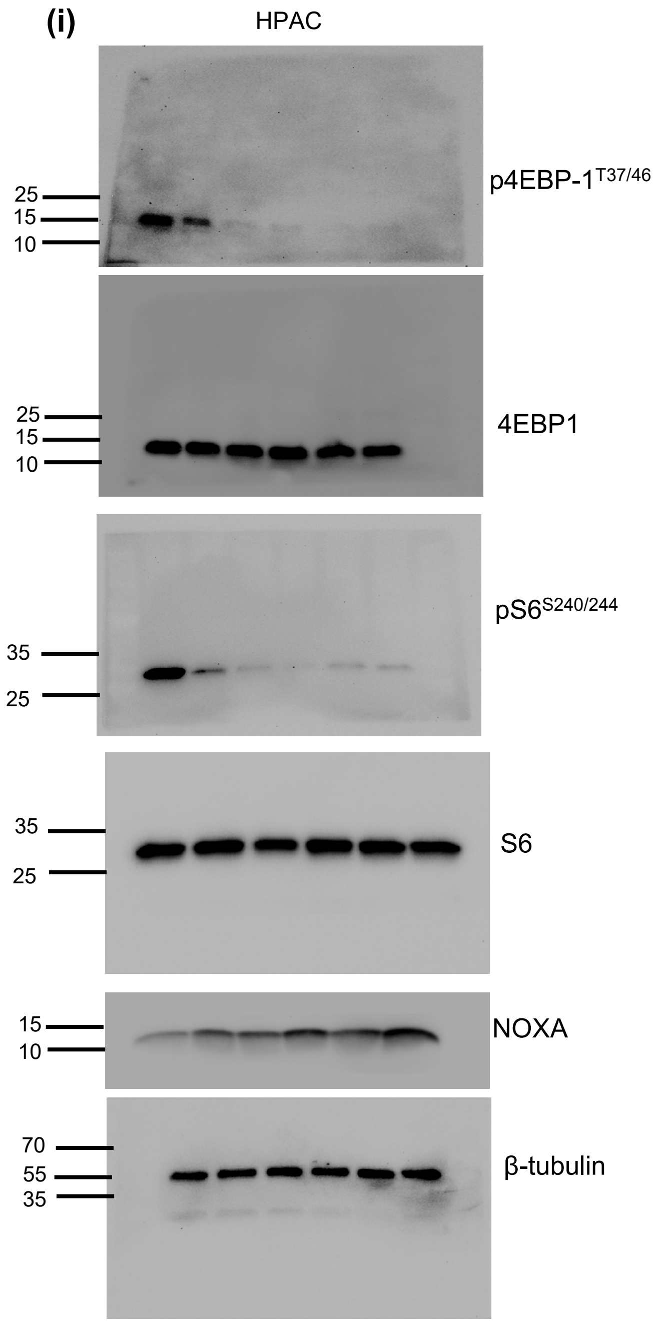
(i)
HPAC
25
15
10
p4EBP-1T37/46
25
15
10
4EBP1
35
25
pS6S240/244
35
25
S6
15
10
NOXA
70
55
35
β-tubulin

Figure 5

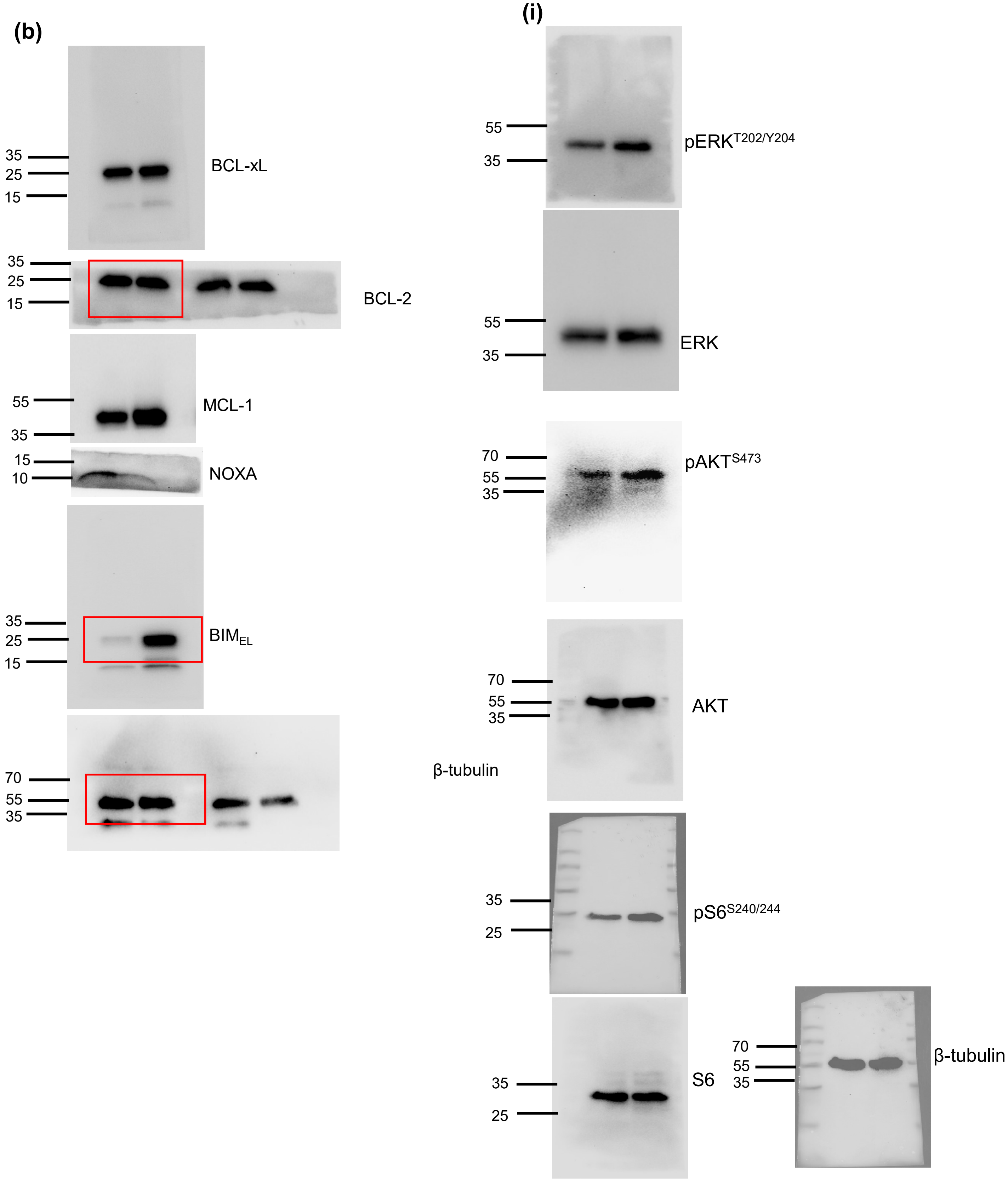

(b)
35
25
15
BCL-xL
35
25
15
BCL-2
55
35
MCL-1
15
10
NOXA
35
25
15
BIMEL
70
55
35
β-tubulin
(i)
55
35
pERKT202/Y204
55
35
ERK
70
55
35
pAKTS473
70
55
35
AKT
35
25
pS6S240/244
35
25
S6
70
55
35
β-tubulin

Figure 5

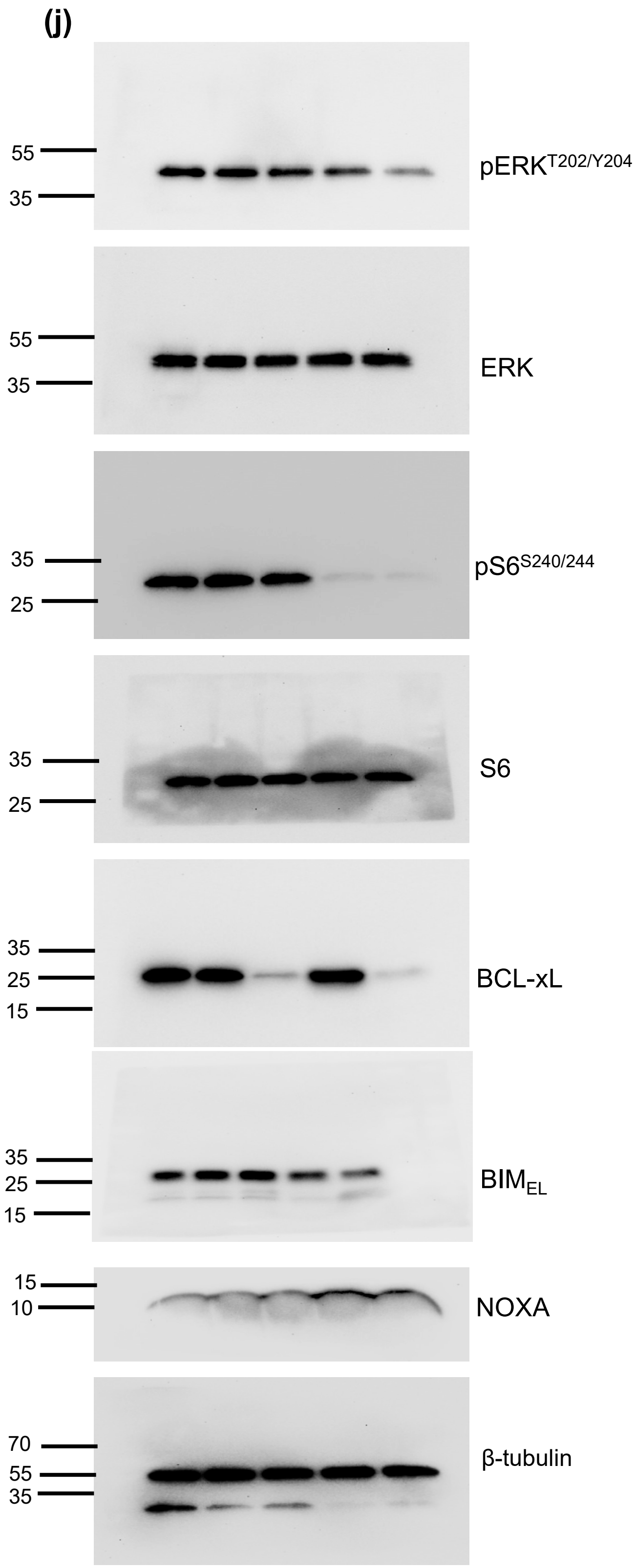
(j)
55
35
pERK^T202/Y204
55
35
ERK
35
25
pS6^S240/244
35
25
S6
35
25
15
BCL-xL
35
25
15
BIM_EL
15
10
NOXA
70
55
35
β-tubulin

Supplementary Figure S1

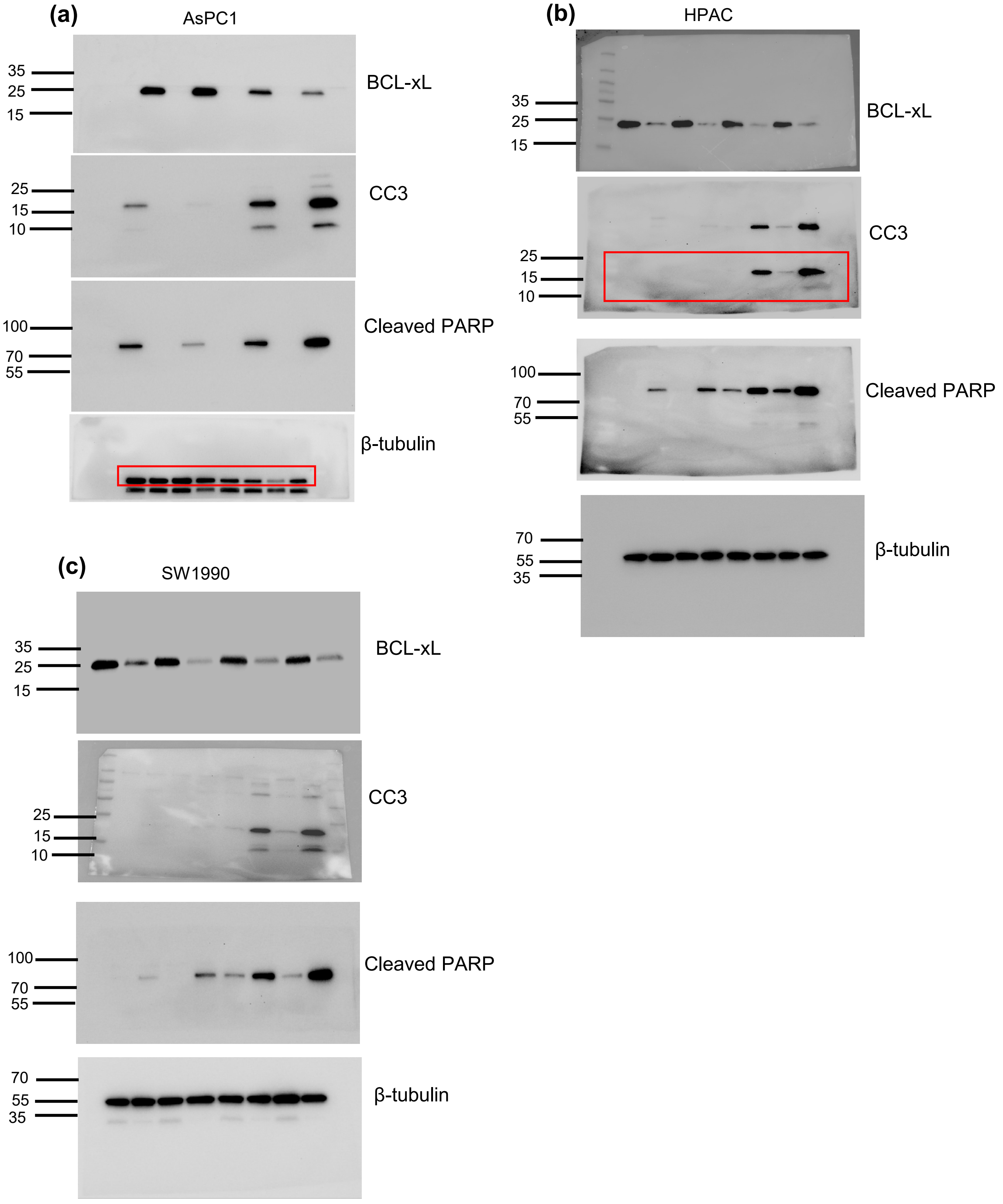

Supplementary Figure S2

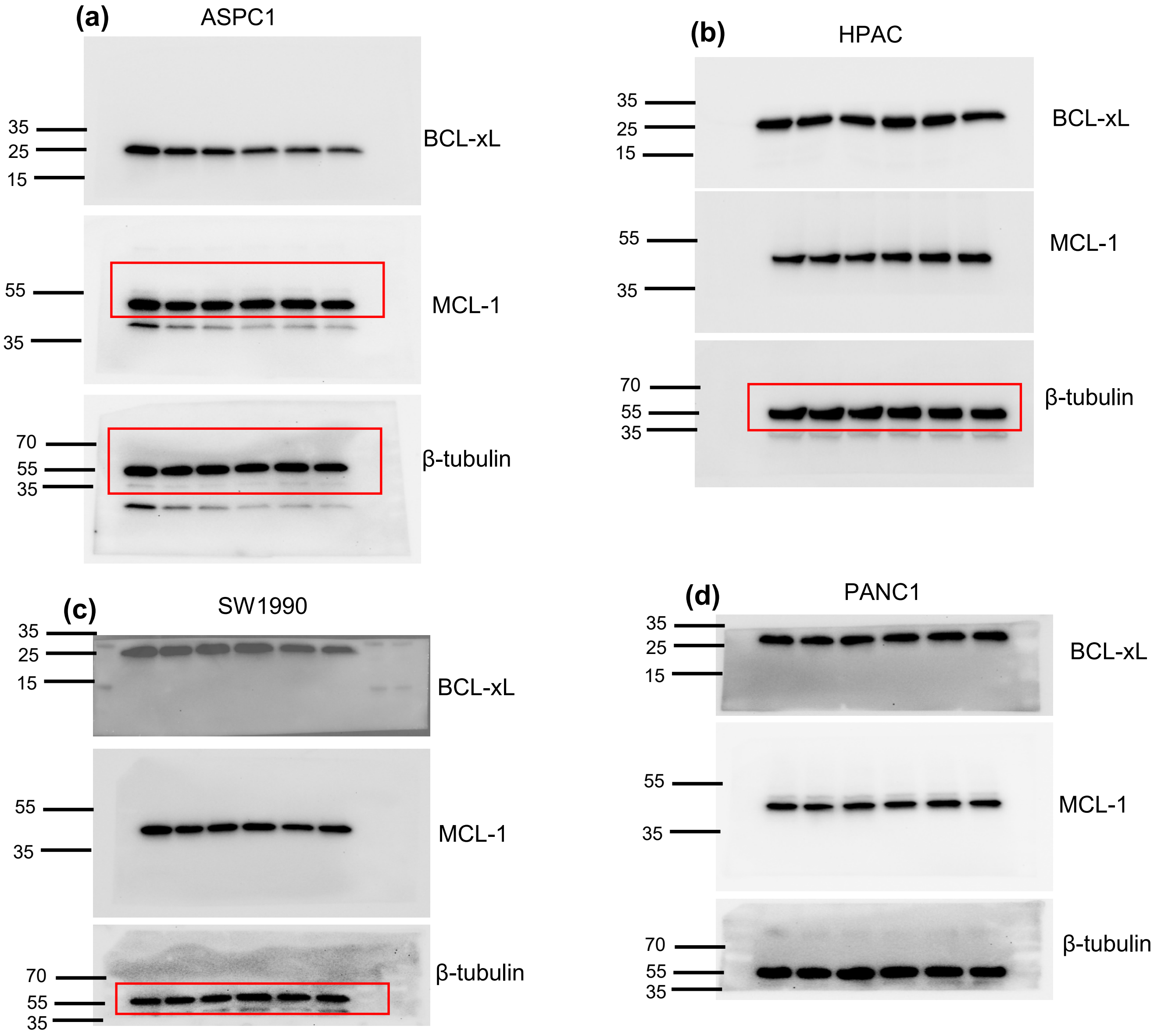

Supplementary Figure S3

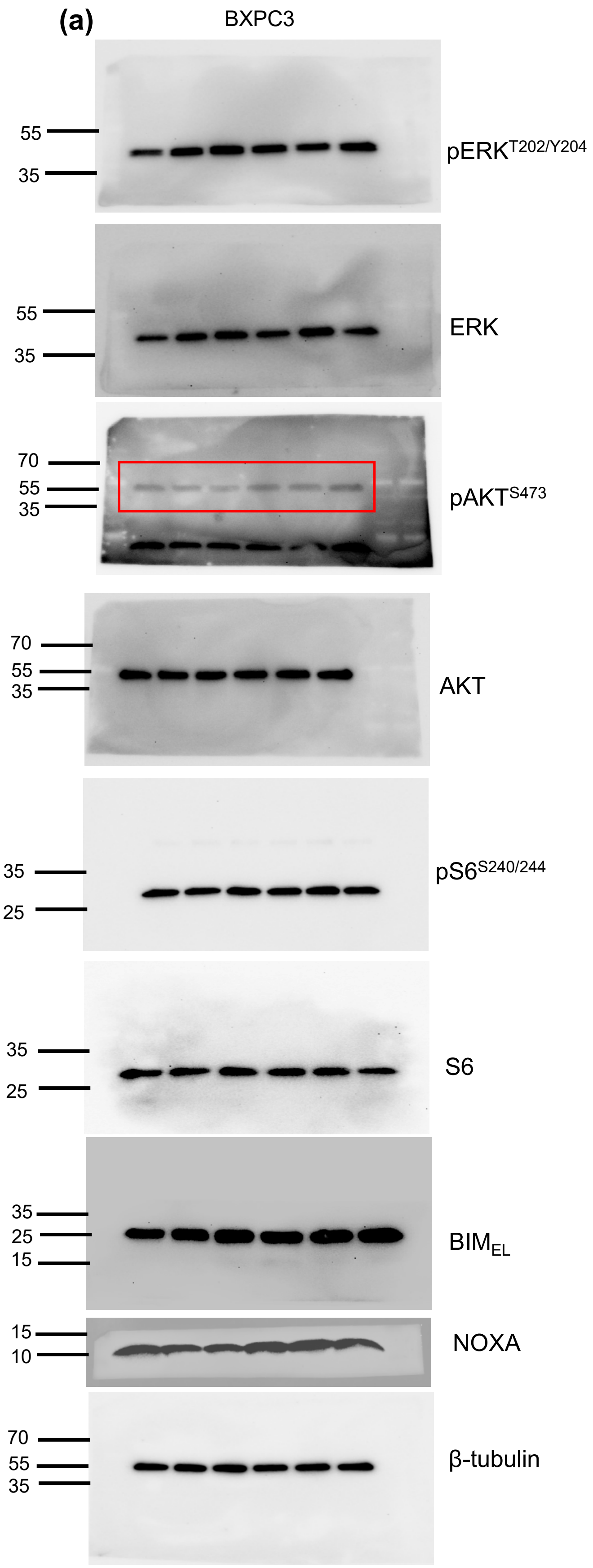

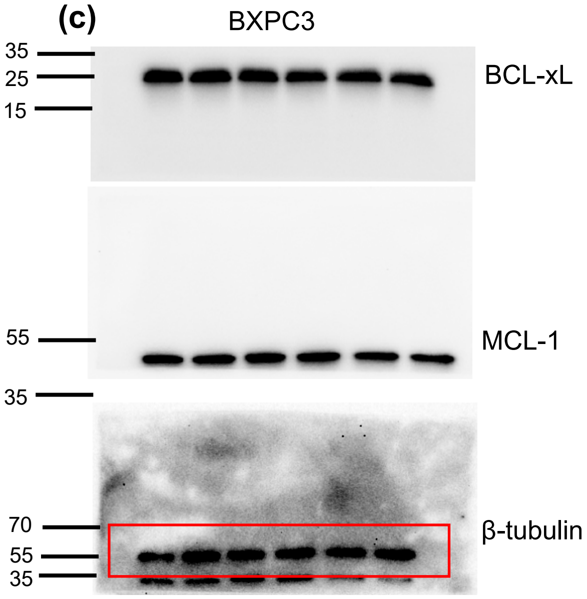

Supplementary Figure S4

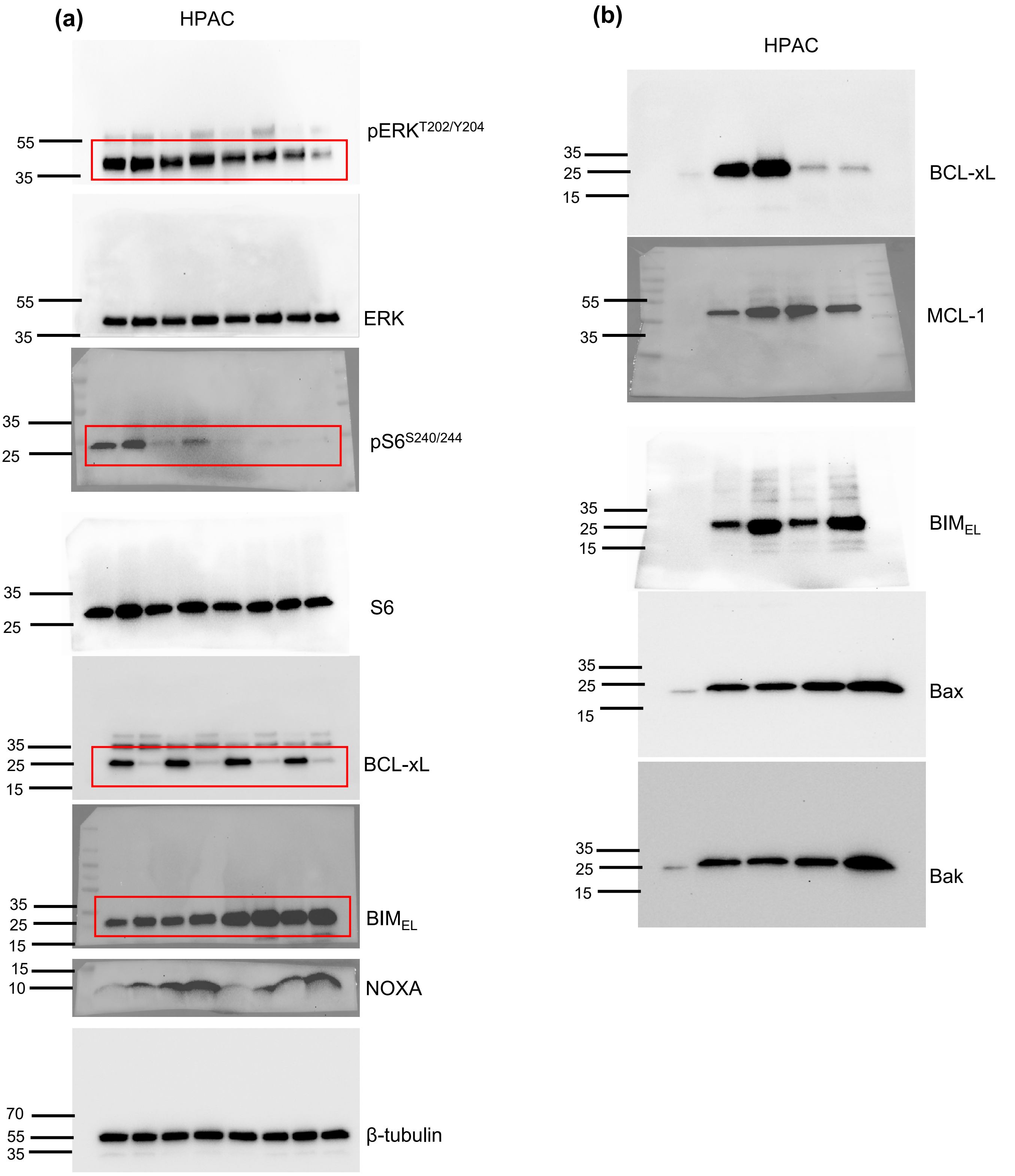

Supplementary Figure S4

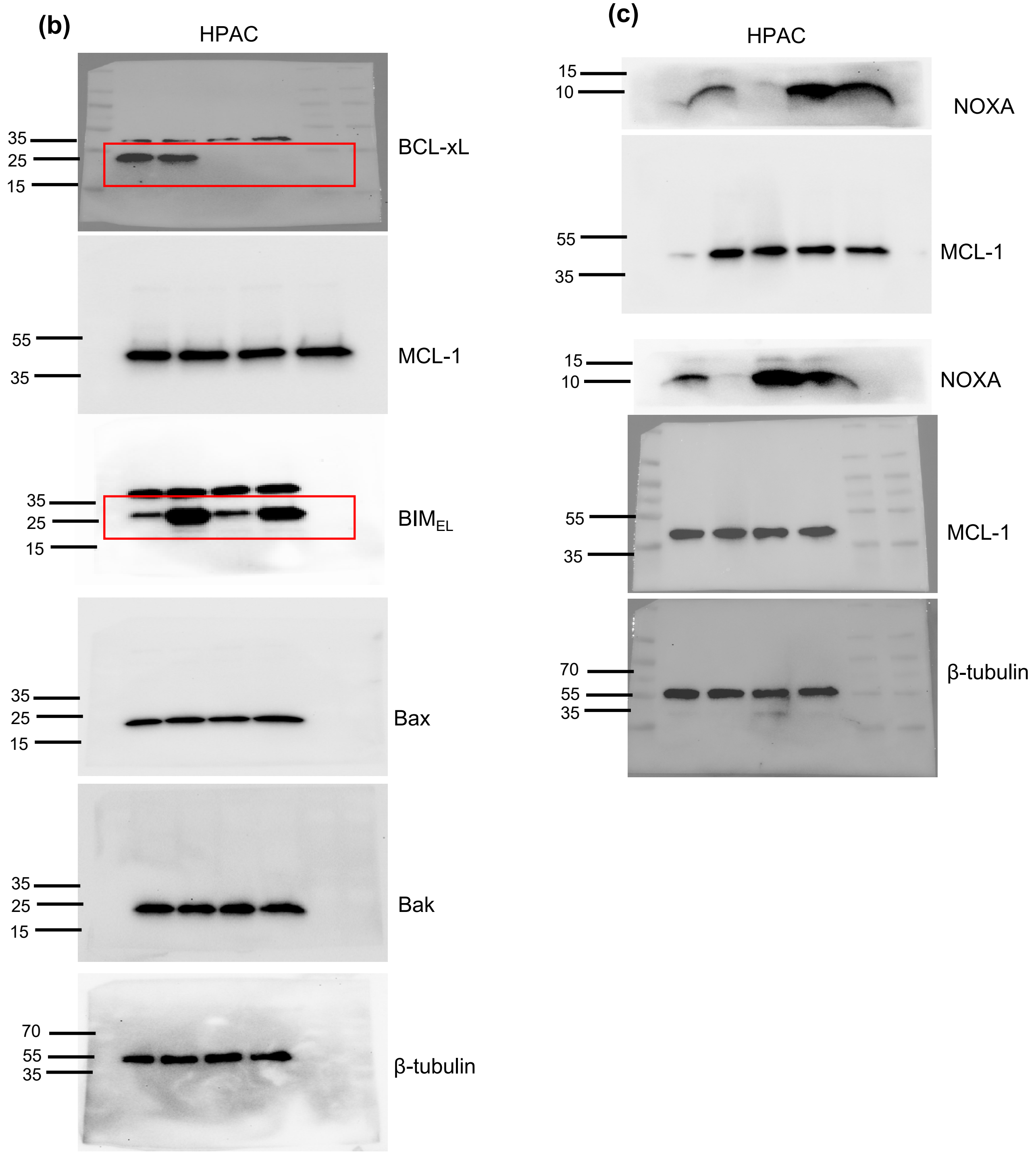

Supplementary Figure S4

(d)

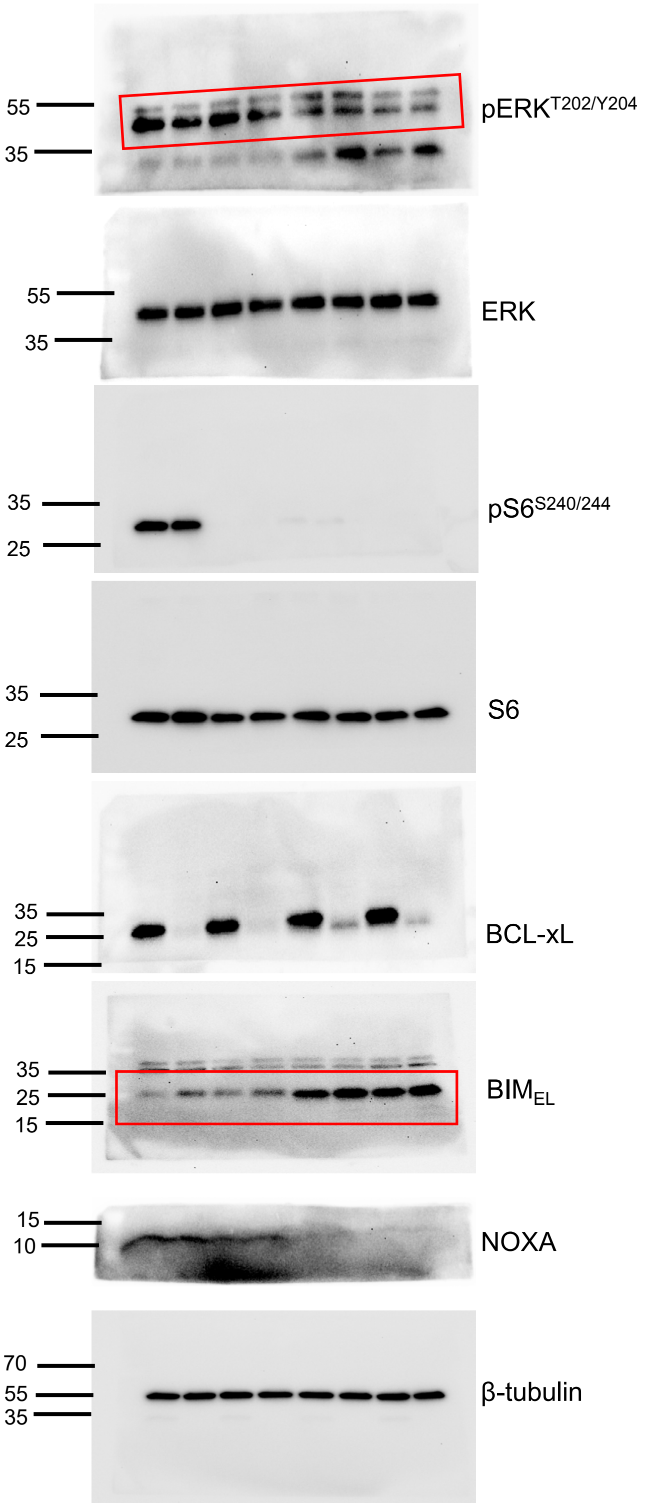

(e)

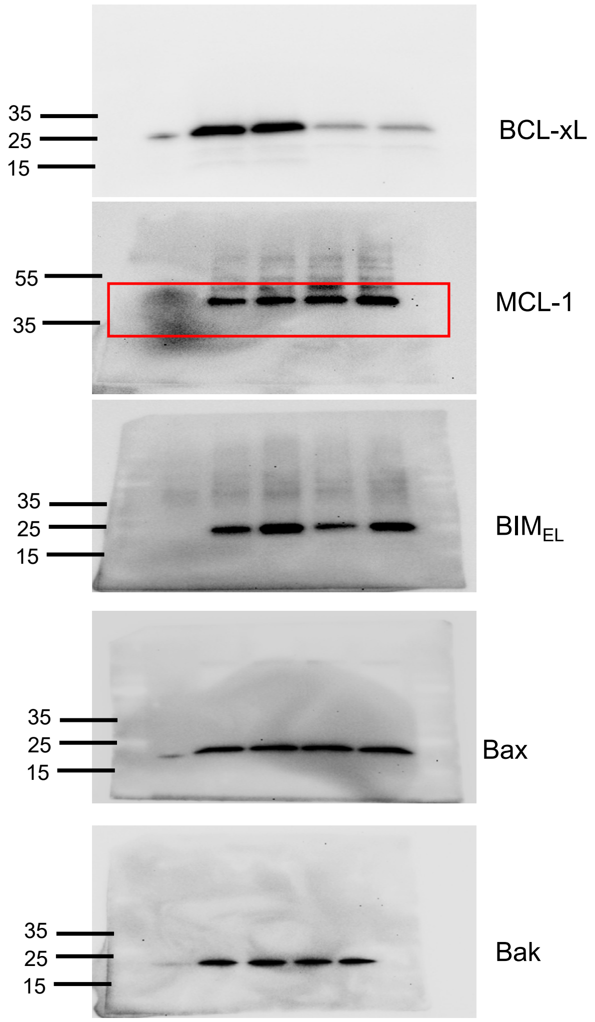

Supplementary Figure S4

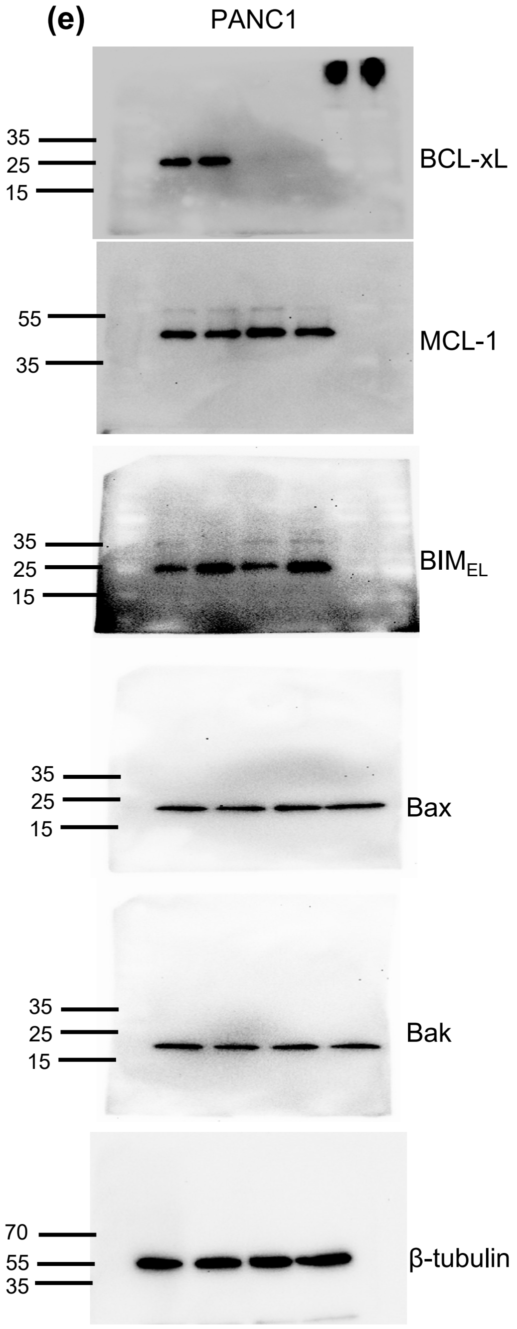

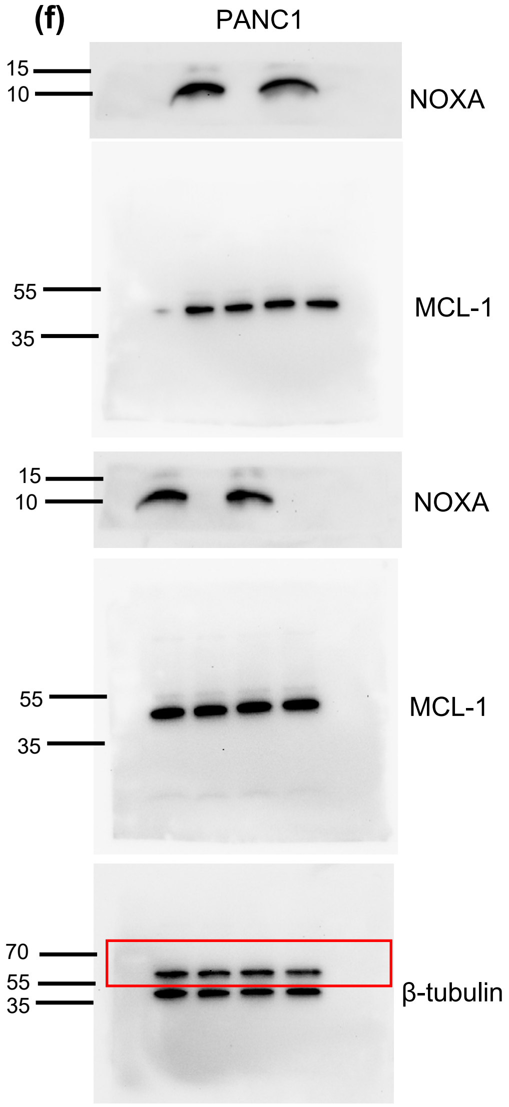

Supplementary Figure S4

(g) AsPC1

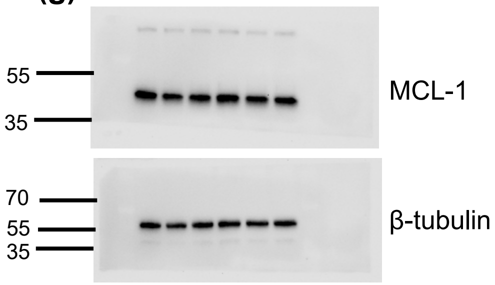

(h) HPAC

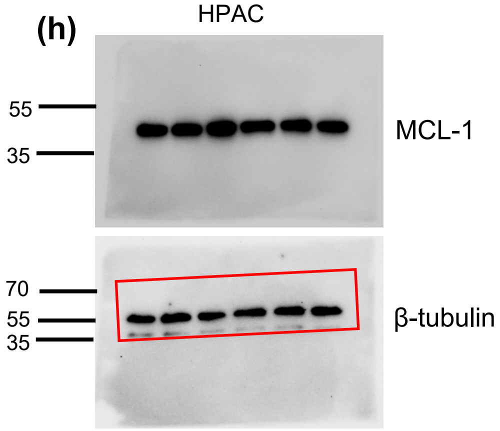

(i) SW1990

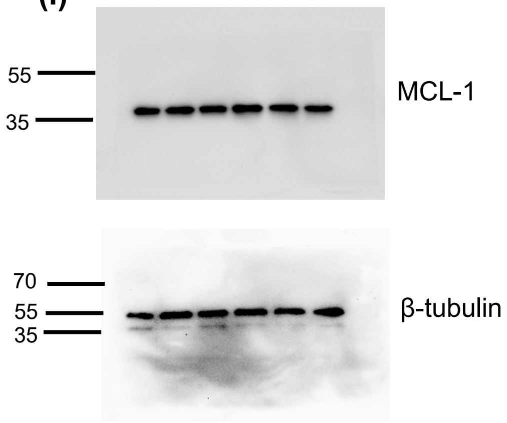

Supplementary Figure S7

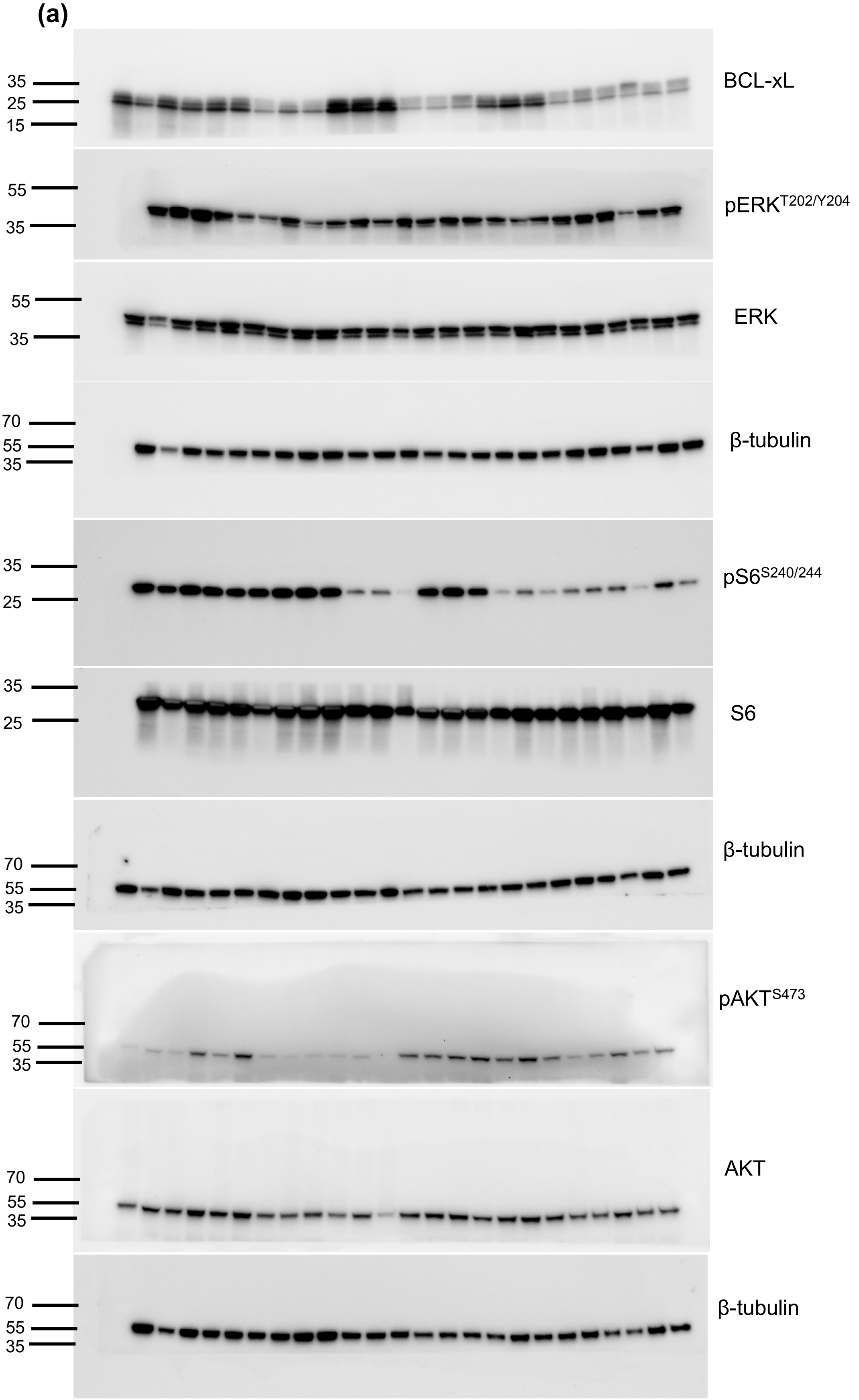

Supplementary Figure S8

(a)

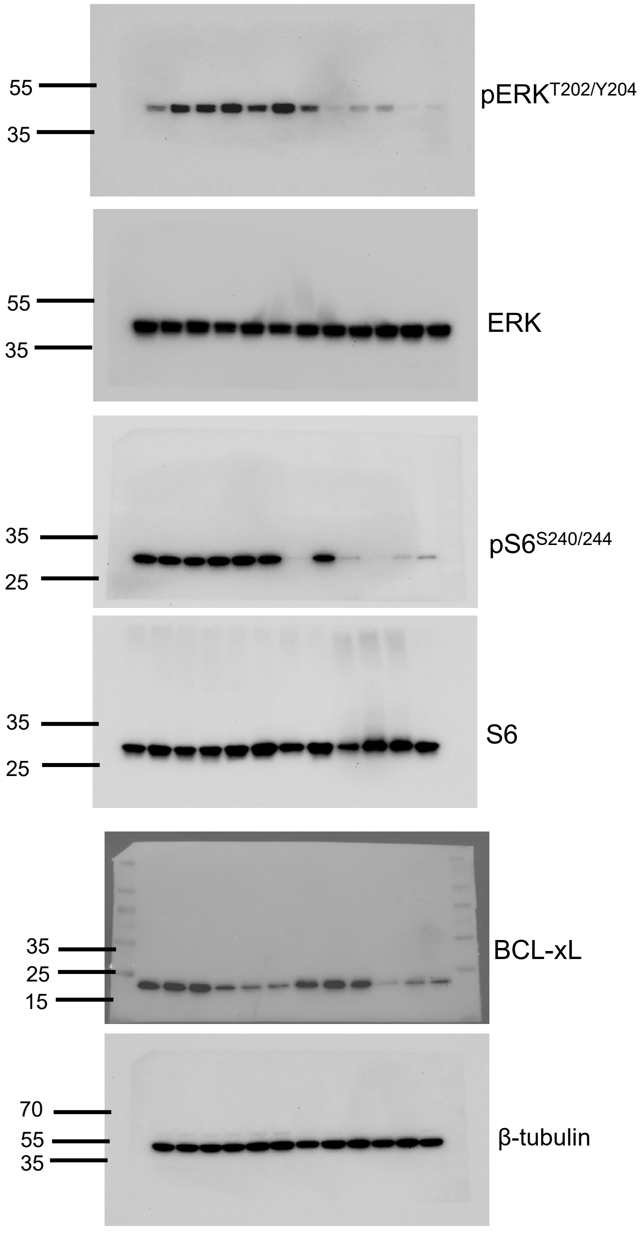

Supplement: Supplementary file 1 [file cancers-18-00920-s001.zip › File S1_Original Western blots-updated.pdf]
