# Supplementary material for: The Combination of a BCL-xL PROTAC and an mTOR Inhibitor Sensitizes Pancreatic Ductal Adenocarcinoma to KRASG12D Inhibitor Treatment
Source: Cancers (Basel). 2026 Mar 12;18(6):920. doi: 10.3390/cancers18060920 (PMC13025216; doi:10.3390/cancers18060920)
Supplement: Supplementary file 1 [file cancers-18-00920-s001.zip › Supplementary Info_Updated.pdf]

SUPPLEMENTARY INFORMATION

The Combination of a BCL-xL PROTAC and an mTOR Inhibitor Sensitizes Pancreatic Ductal Adenocarcinoma to KRAS^{G12D} Inhibitor Treatment

Javed Miyan [1], Vignesh Vudatha [2], Lin Cao [1], Peiyi Zhang [3], Guangrong Zheng [3], Lei Zheng [4], Jose Trevino [2], Daohong Zhou [1,4,*] and Sajid Khan [1,4,*]

[1] Department of Biochemistry & Structural Biology, The University of Texas at San Antonio, UT Health San Antonio, 7703 Floyd Curl Drive, San Antonio, TX 78229, USA
[2] Department of Surgery, Virginia Commonwealth University School of Medicine, Richmond, VA 23298, USA
[3] Department of Medicinal Chemistry, College of Pharmacy, University of Florida, Gainesville, FL 32610, USA
[4] Mays Cancer Center, The University of Texas at San Antonio, UT Health San Antonio, 7703 Floyd Curl Drive, San Antonio, TX 78229, USA
* Correspondence: zhoud@uthscsa.edu (D.Z.); khans10@uthscsa.edu (S.K.); Tel.: +1-210-450-3875 (D.Z.); +1-210-450-7888 (S.K.)

SUPPLEMENTARY FIGURES

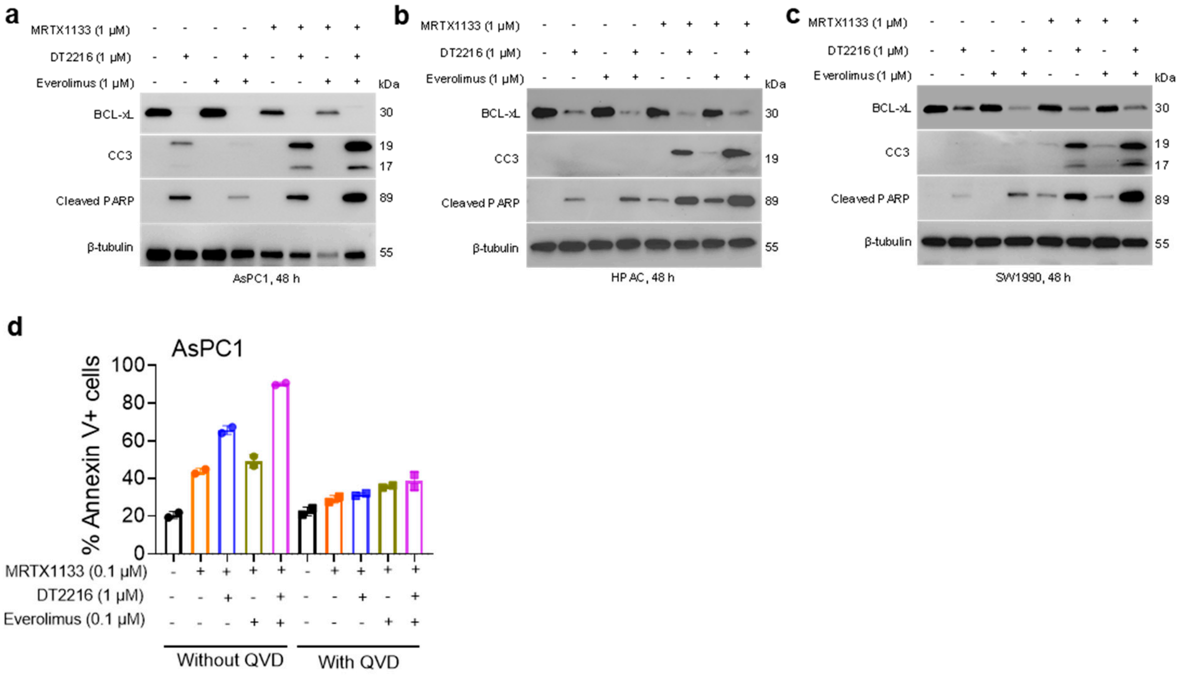

Supplementary Figure S1. **a–c.** Immunoblot analyses of BCL-xL, cleaved caspase-3 (CC3) and cleaved PARP in AsPC1 (a), HPAC (b) and SW1990 cells (c) after they were treated with MRTX1133, DT2216, everolimus and their combinations as indicated for 48 h. β-tubulin was used as an equal loading control in all immunoblots. **d**. Percentage annexin V+ apoptotic population without and with 10 μM QVD-pretreated AsPC1 cells after treatment with MRTX1133 and its combinations with DT2216 and/or everolimus at indicated concentrations for 48 h.

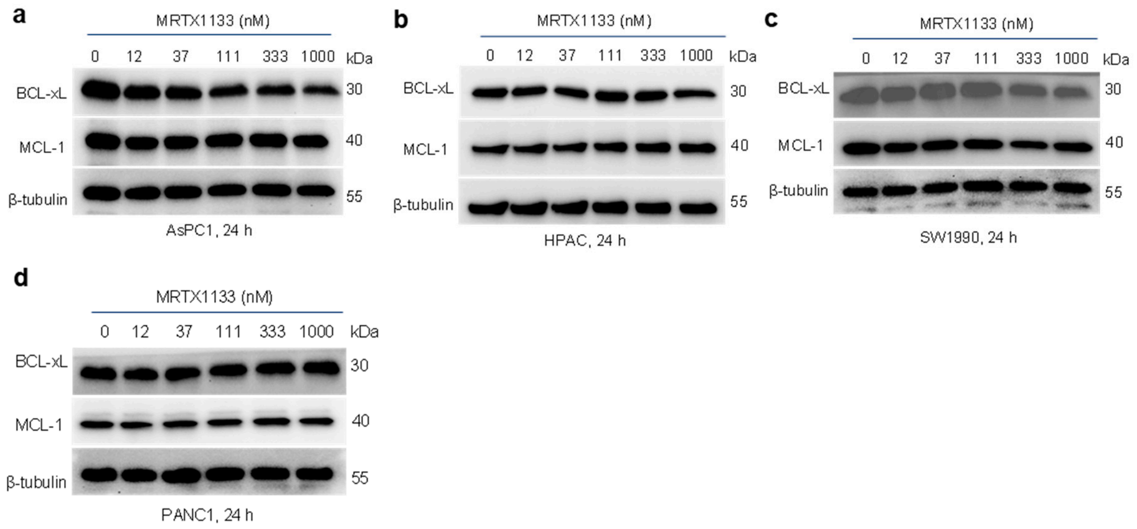

Supplementary Figure S2. **a-d**. Immunoblot analyses of BCL-xL and MCL-1 in AsPC1 (a), HPAC (b), SW1990 (c), and PANC1 (d) cells after they were treated with indicated concentrations of MRTX1133 for 24 h. The β-tubulin was used as an equal loading control.

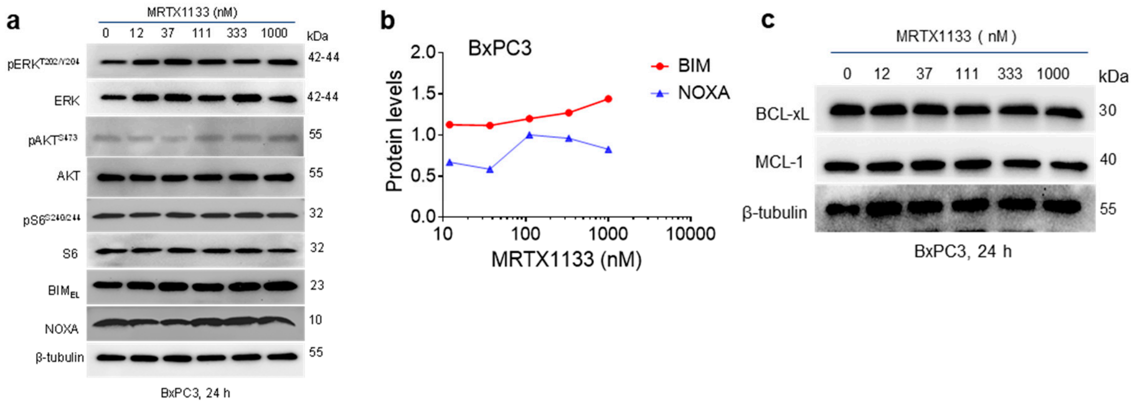

Supplementary Figure S3. **a.** Immunoblot analyses of phosphorylated- and total- ERK, AKT and S6, BIM extra-long isoform (BIM_{EL}) and NOXA in AsPC1 cells after they were treated with indicated concentrations of MRTX1133 for 24 h. **b.** Densitometric analysis of BIM_{EL} and NOXA immunoblots in AsPC1 cells. **c**. Immunoblot analyses of BCL-xL and MCL-1 in BxPC3 cells. The β-tubulin was used as an equal loading control.

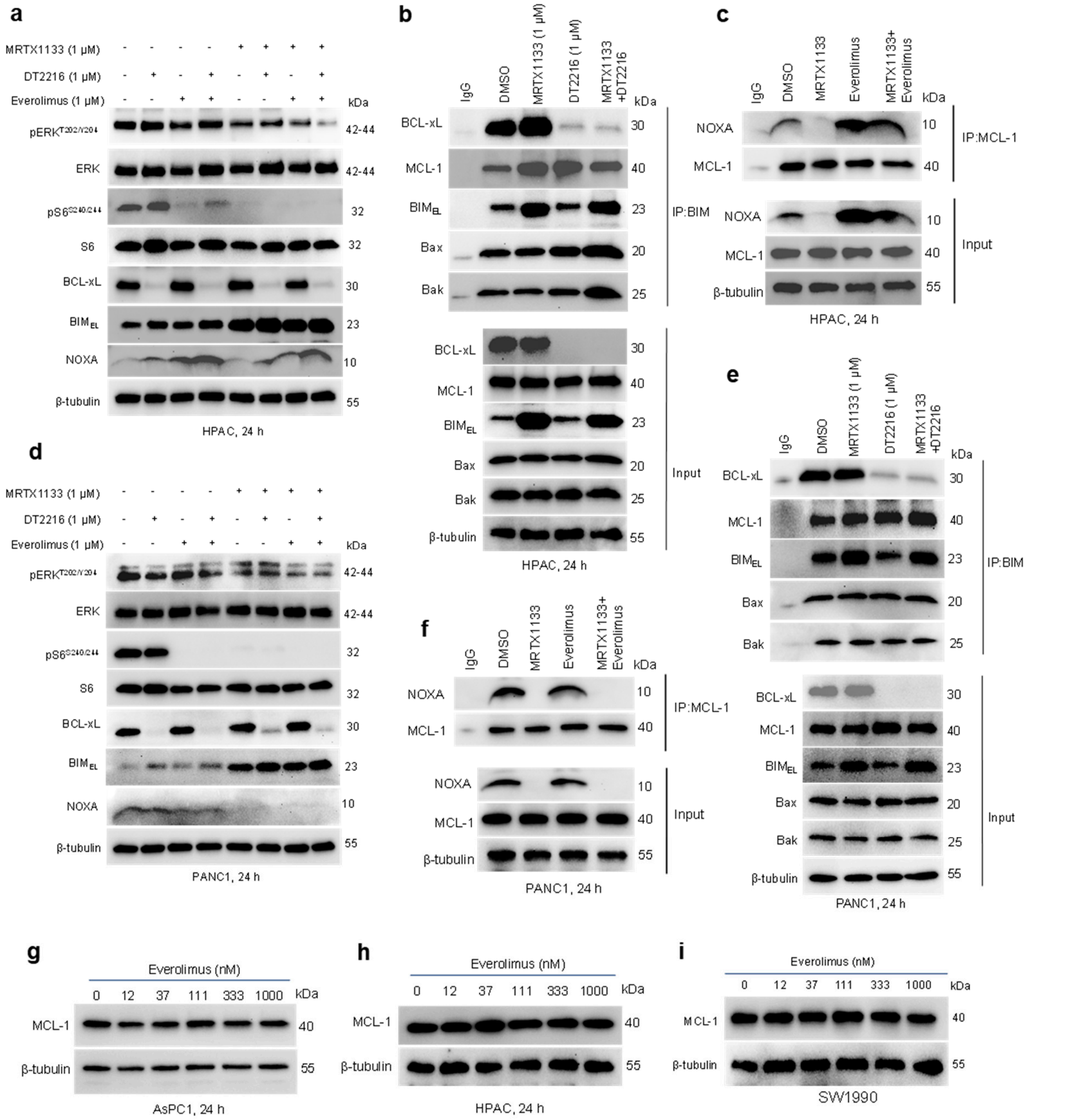

Supplementary Figure S4. a-c. a, d. Immunoblot analyses of phosphorylated- and total-ERK and S6, BCL-xL, BIM and NOXA in HPAC (a) and PANC1 (d) cells after they were treated with MRTX1133, DT2216, everolimus and their combinations as indicated for 24 h. **b, e**. Immunoprecipitation analysis of BIM in HPAC (b) and PANC1 (e) cells after they were treated with DMSO, MRTX1133, DT2216 or MRTX1133+DT2216 for 24 h, and the

immunoprecipitated as well as input samples were subjected to immunoblot analysis of BCL-X_L, MCL-1, and BIM. **c, f**. Immunoprecipitation analysis of MCL-1 in HPAC (c) and PANC1 (f) cells after they were treated with DMSO, MRTX1133, everolimus or MRTX1133+everolimus for 24 h, and the immunoprecipitated as well as input samples were subjected to immunoblot analysis of NOXA and MCL-1. **g-i.** Immunoblot analyses of MCL-1 in AsPC1 (g), HPAC (h) and SW1990 cells (i) after they were treated with indicated concentrations of everolimus for 24 h. The β-tubulin was used as an equal loading control.

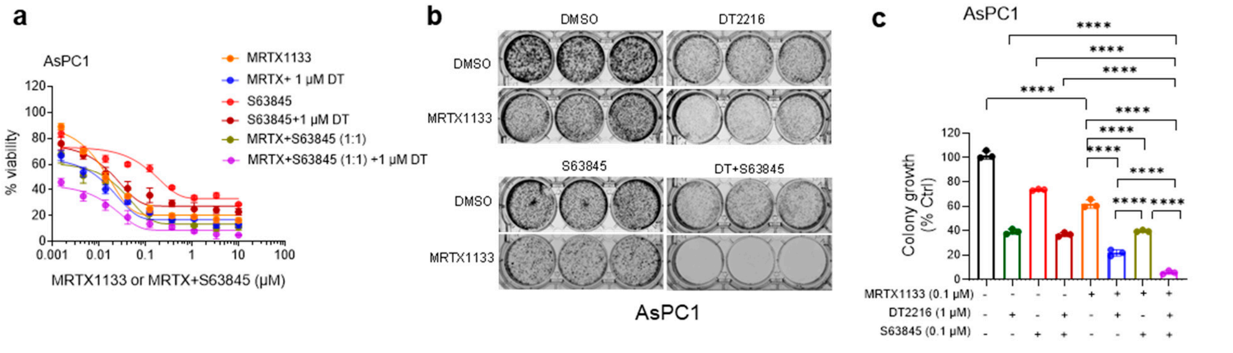

Supplementary Figure S5. a. Viability of parental AsPC1 cells after they were treated with increasing concentrations of MRTX1133, S63845 alone and/or with 1 μM of DT2216 and/or equimolar (1:1) ratio of S63845 for 72 h. Data are presented as ± SD (n= 3 replicate cell cultures). First points in MRTX + 1 μM DT2216, S63845 + 1 μM DT2216 and MRTX + S63845 (1:1) + 1 μM DT2216 represent 1 μM DT2216 alone. **b.** Colony images of AsPC1 cells after they were treated with MRTX1133, DT2216, S63845 and their combinations as indicated for 14 days followed by crystal violet staining. **c.** Colorimetric measurement of colony growth in AsPC1 cells. Data are presented as mean ± SD (n =3 cell culture replicates). Statistical significance was determined by one-way ANOVA and Tukey's multiple comparisons test, where ****p <0.0001.

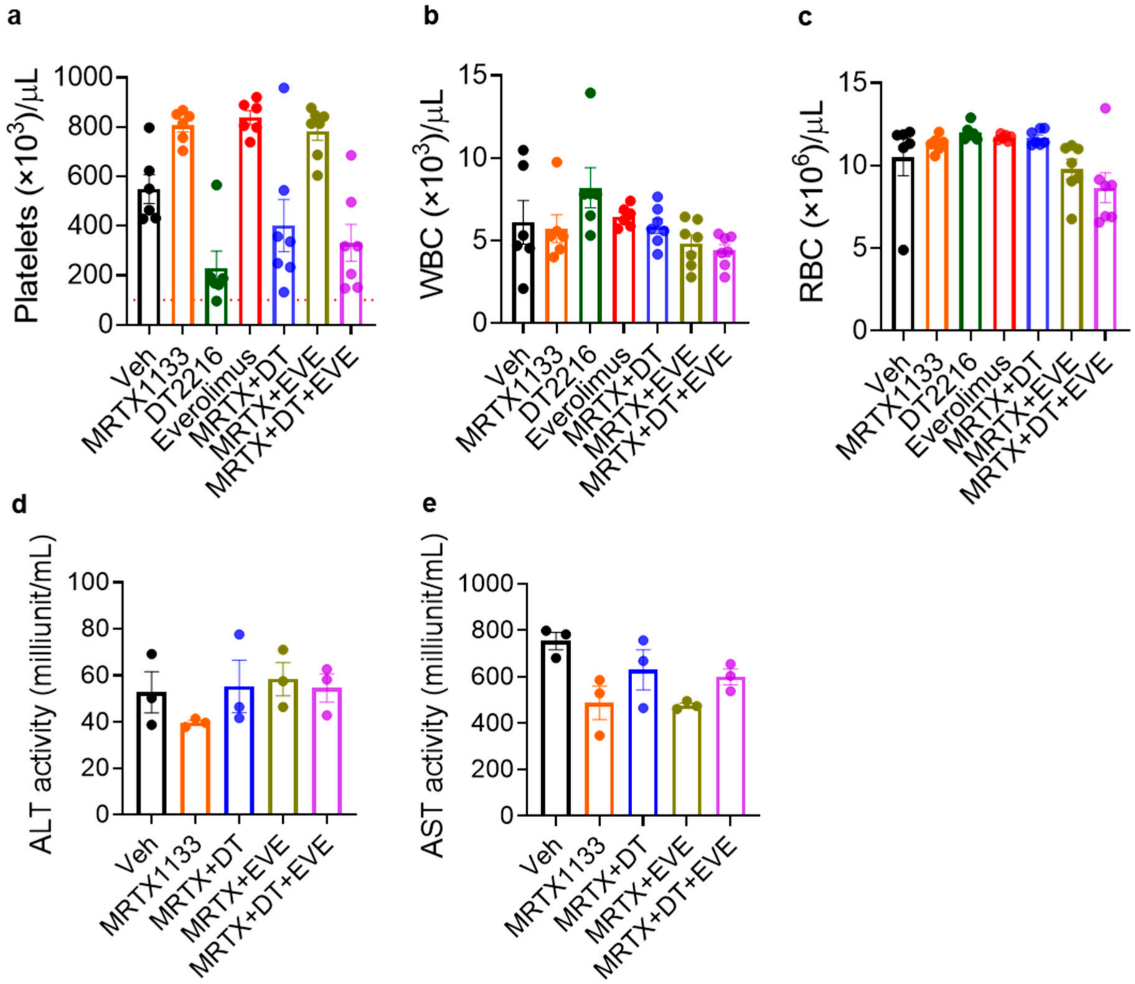

Supplementary Figure S6. a-c. Enumeration of platelets (a), WBCs (b), and RBCs (c) 24 h after the final dose of DT2216. Data are presented as mean ± SEM (n = 6-7 mice per group). **d, e**. ALT (d) and AST (e) activities in mouse serum as measured 24 h after final dose of DT2216. Data are presented as mean ± SEM (n = 3 mice per group).

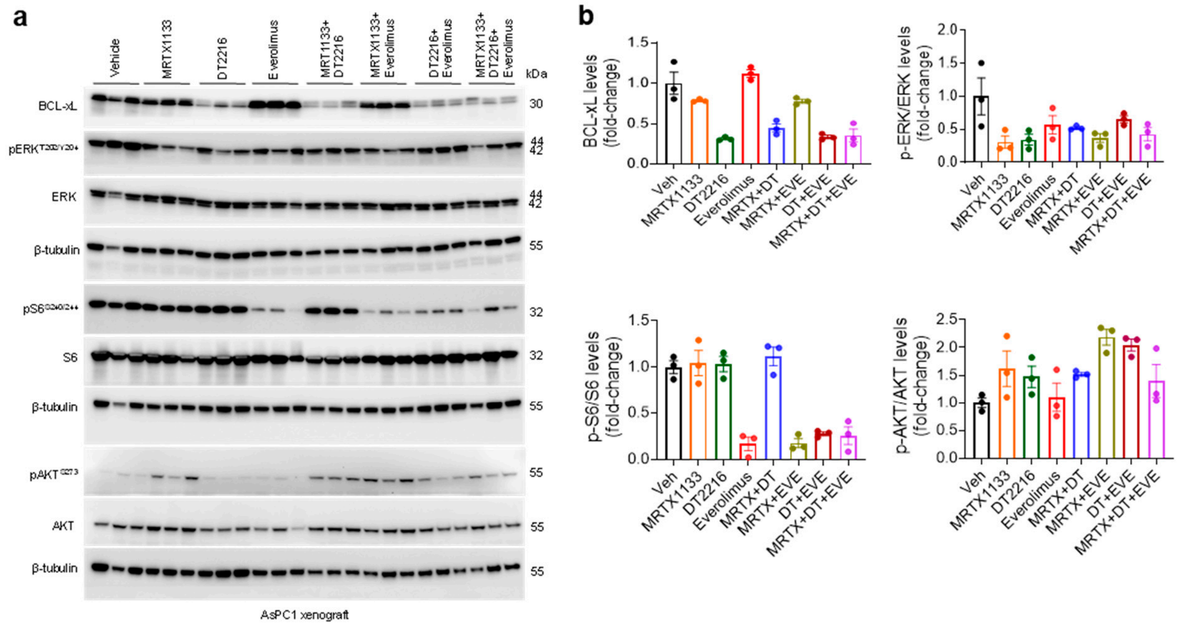

Supplementary Figure S7. The MRTX1133, DT2216 and everolimus demonstrate their respective target inhibition in AsPC1 xenografts. a. Immunoblot analyses of BCL-xL, phosphorylated- and total- ERK, S6, and AKT in AsPC1 xenograft mice after they were treated with vehicle, MRTX1133 (MRTX, 3 mg/kg, b.i.d, Day 1-5, i.p.), DT2216 (DT, 15 mg/kg, Day 1 and Day 5, i.p.), everolimus (EVE, 2.5 mg/kg, Day 1 and Day 5, p.o.) or their combinations as indicated and tumors were harvested 24 h after 2nd dose of DT2216 i.e., Day 6. The β-tubulin was used as an equal loading control. **b.** Densitometric analysis of immunoblots as in **a**. Data are presented as mean ± SEM (n = 3 mice per group).

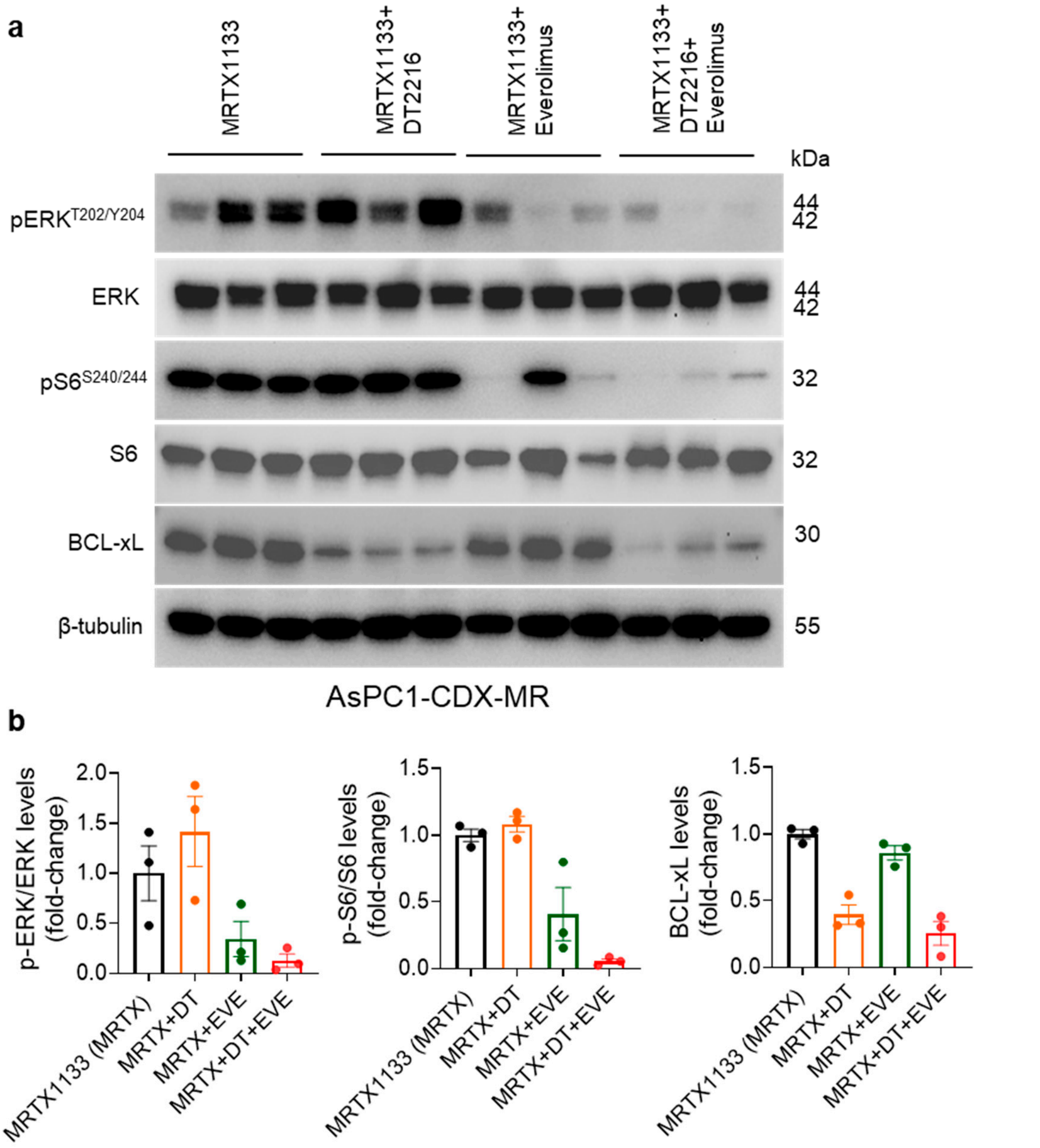

Supplementary Figure S8. a. Immunoblot analyses of phosphorylated- and total- ERK and S6, BCL-xL, BIM and NOXA in AsPC1-CDX-MR tumors after indicated treatments. **b**. Densitometric analysis of immunoblots as in **a**. Data are presented as mean ± SEM (n = 3 mice per group).

Supplementary Table S1. Antibodies used in immunoblotting.

Antibody	Clone	Antibody	Catalog	RRID	Concentration
p-ERK T202/Y204	_	Rabbit IgG	9101	AB_331646	1:1000
ERK	_	Rabbit IgG	9102	AB_330744	1:1000
p-AKT S473	193H12	Rabbit IgG	4058	AB_331168	1:1000
AKT	_	Rabbit IgG	9272	AB_329827	1:1000
p-S6 S240/244	_	Rabbit IgG	2215	AB_331682	1:1000
S6	5G10	Rabbit IgG	2217	AB_331355	1:1000
BIM	C34C5	Rabbit IgG	2933	AB_1030947	1:1000
NOXA	D8L7U	Rabbit IgG	14766	AB_2798602	1:1000
p-4EBP1 T37/46	236B4	Rabbit IgG	2855	AB_560835	1:1000
4EBP1	53H11	Rabbit IgG	9644	AB_2097841	1:1000
BCL-xL	_	Rabbit IgG	2762	AB_10694844	1:1000
BCL-2	D55G8	Rabbit IgG	4223	AB_1903909	1:1000
MCL-1	D35A5	Rabbit IgG	5453	AB_10694494	1:1000
BAX	_	Rabbit IgG	2772	AB_10695870	1:1000
BAK	D4E4	Rabbit IgG	12105	AB_2716685	1:1000
Cleaved PARP	D64E10	Rabbit IgG	5625	AB_10699459	1:1000
Cleaved caspase-3	_	Rabbit IgG	9661	AB_10699459	1:1000
β-tubulin	_	Rabbit IgG	2146	AB_2210545	1:3000
Secondary antibody	_	Anti-rabbit	7074	AB_2099233	1:3000

Footnotes: All the antibodies were purchased from Cell Signaling Technology, Danvers, MA.
